# A new sphenodontian (Diapsida: Lepidosauria) from the Upper Triassic (Norian) of Germany and its implications for the mode of sphenodontian evolution

**DOI:** 10.1186/s12862-024-02218-1

**Published:** 2024-03-16

**Authors:** Lisa S. Freisem, Johannes Müller, Hans-Dieter Sues, Gabriela Sobral

**Affiliations:** 1 Department of Earth and Planetary Sciences, Yale University, New Haven, CT 06520 USA; 2 Museum für Naturkunde Berlin, Leibniz-Institut für Evolutions- Und Biodiversitätsforschung, Invalidenstraße 43, Berlin, 10115 Germany; 3 Department of Paleobiology, National Museum of Natural History, Smithsonian Institution, MRC 121, Washington, DC USA; 4 Staatliches Museum für Naturkunde Stuttgart, Rosenstein 1, Stuttgart, 70191 Germany

**Keywords:** Sphenodontia, Rhynchocephalia, Triassic, Norian, Lepidosauromorph, Skull

## Abstract

The Arnstadt Formation of Saxony-Anhalt, Germany has yielded some of Germany’s most substantial finds of Late Triassic tetrapods, including the sauropodomorph *Plateosaurus* and the stem-turtle *Proganochelys quenstedti*. Here, we describe an almost complete skull of a new sphenodontian taxon from this formation (Norian, 227–208 Ma), making it the oldest known articulated sphenodontian skull from Europe and one of the oldest in the world. The material is represented by the dermal skull roof and by the complete maxilla and temporal region, as well as parts of the palate, braincase, and lower jaw. A phylogenetic assessment recovers it as a basal sphenodontian closely related to *Planocephalosaurus* *robinsonae* and to Eusphenodontia, making it the earliest-diverging sphenodontian known with an articulated skull. Its cranial anatomy is generally similar to the well-known *Diphydontosaurus avonis* from the Rhaetian of England, showing that this successful phenotype was already established in the clade around 10 myr earlier than assumed. An analysis of evolutionary change rates recovers high rates of evolution in basal sphenodontians, with decreasing rates throughout the evolution of the group. However, contrary to previous studies, reversals in this trend were identified, indicating additional peaks of evolutionary change. These results improve our understanding of the early sphenodontian diversity in Europe, providing critical information on evolutionary trends throughout the history of the clade and sparking renewed interest in its evolution.

**Supplementary Information:**

The online version contains supplementary material available at 10.1186/s12862-024-02218-1.

## Background

During the Triassic Period, the major living lineages of reptiles, including archosaurs [1, 2], squamates [3] and rhynchocephalians [4], began to diversify. Rhynchocephalians are represented in the modern fauna solely by the tuatara *Sphenodon punctatus*, but they were the dominant lepidosaur group during the Triassic and Jurassic [5–12]. Mesozoic rhynchocephalians were trophically diverse [7], and included specialised herbivores (*Toxolophosaurus cloudi,* [13]; *Priosphenodon avelasi* [14]), insectivores (*Gephyrosaurus bridensis,* [15]; *Diphydontosaurus avonis* [11]), the possibly durophagous sapheosaurids (e.g., *Oenosaurus muehlheimensis* [16]), as well as supposedly venomous predators (*Sphenovipera jimmysjoyi* [17]). Their disparity also manifested in their body size variation, which ranged over one order of magnitude [7]. Most of this disparity is contained within Sphenodontia (= ”Sphenodontida” sensu [18] and as applied in [19], all rhynchocephalians more closely related to *Sphenodon punctatus* than *Gephyrosaurus bridensis*). Although they were a dominant component of the Mesozoic fauna, Laurasian sphenodontian diversity and abundance rapidly decreased during the Early Cretaceous [10, 20], possibly due to the changing climate during the fragmentation of Pangea in this period [16]. South American sphenodontians remained abundant until the Campanian and were only replaced by squamates during the Paleogene after their diversity had already declined in the Late Jurassic and Early Cretaceous [10, 14, 21]. The complex evolutionary history of sphenodontians, including the high diversity in the Triassic and multiple occurrences of highly specialised taxa (including at least two aquatic radiations [22]), has made them subject of several evolutionary rates studies. A recent investigation [21] showed that body size evolutionary rates were a lot higher in extinct lepidosaurian clades including rhynchocephalians compared to squamates during the Mesozoic, suggesting advantages of slow evolution for long-term survival. Within Sphenodontia, however, analyses produced different results. While some studies yielded a steady decrease in change rates throughout Sphenodontia [23], or decreasing, but more heterogeneous rates within the clade and higher rates on terminal than internal branches [24], an approach focusing on body size evolutionary rates [21] revealed increasing rates within Mesozoic Sphenodontia throughout time. Many sphenodontians have been discovered in the Triassic and Jurassic formations of Germany, including *Polysphenodon muelleri* [25, 26], *Oenosaurus muehlheimensis* [16], *Vadasaurus herzogi* [27], and *Palaeopleurosaurus posidoniae* [26], as well as the basal rhynchocephalian *Wirtembergia hauboldae* [19]. The steady increase in information on early sphenodontians is a valuable resource for understanding the evolutionary dynamics of the clade and might offer new insights into the evolutionary rates within it.

Here we describe a new early-diverging sphenodontian based on an almost complete skull with an incomplete lower jaw in a block of sedimentary rock found by Werner Janensch during the excavation of a skeleton of the sauropodomorph *Plateosaurus* in 1928. Since it is fully enclosed in sediment, this skull went unnoticed until a recent re-evaluation of *Elachistosuchus huenei* MB.R.4520.2 by [28] using µCT scanning. The discovery of this new sphenodontian gives new insights into the early evolution of Sphenodontia and changes in the tempo of their morphological disparification.

## Systematic palaeontology

Lepidosauria Haeckel, 1866 sensu Evans 1984.

Rhynchocephalia Günther, 1867 sensu Gauthier et al., 1988.

Sphenodontia Williston, 1925 sensu Gauthier et al., 1988.

*Parvosaurus harudensis*, gen. et sp. nov.

LSID urn:lsid:zoobank.org:pub:B21E64B7-D7FC-4C17-93B3-359D4D27CFF5

### Etymology

*Parvus*, meaning small in Latin, refers to the small size of the new taxon (total skull length of 16 mm), *saurus* meaning lizard in Latin. The species name *harudensis* is dedicated to the Harz Mountains in Germany (harud being a historic name for Harzgau, including Halberstadt) where the specimen was discovered.

### Holotype

MB.R.4520.2 (Museum of Natural History, Berlin, Germany), a nearly complete skull, fully encased in sediment. It preserves most of the dermal skull roof, both nasals, premaxillae and maxillae, complete right and fragmented left parietal, postorbital, jugal and squamosal, fragmentary palate, a right maxilla and a left dentary, including maxillary as well as mandibular teeth. The braincase is present, but it is not well enough preserved to distinguish between elements.

### Locality and horizon

Brick-clay pit along the present-day highway B79 between Halberstadt and Quedlinburg, on the south-eastern edge of Halberstadt, Saxony-Anhalt, Germany; Arnstadt Formation, “Steinmergelkeuper”, Norian, Late Triassic (227–208 Ma); after a µCT scan in 2013, the holotype of *Parvosaurus harudensis* was discovered inside the sediment block with the bones of the holotype of *Elachistosuchus huenei* MB.R.4520.2 (Museum für Naturkunde, Berlin, Germany), which was collected by Werner Janensch in 1928.

### Diagnosis

*Parvosaurus harudensis* differs from other sphenodontians in the following combination of features: premaxilla with long ascending process reaching almost to a third of the anteroposterior length of the nasal; maxilla with long posterior process reaching up to the middle of the jugal body; dentary with prominent coronoid process; nasals rectangular in outline with anteroposteriorly parallel sutures to prefrontals; postfrontal with well-developed posterior process extending posteriorly beyond the fronto-parietal suture; elongated pineal foramen bounded only by the parietals; posterior border of the parietals strongly incised, forming an angle between posterolateral processes of 110°; posterior process of postorbital at least twice as long as ventral process; medial process of postorbital underlapping lateral process of postfrontal; most of the margin of the supratemporal fenestra formed by parietal and postorbital; maxillary teeth small and pin-like anteriorly, taller and conical posteriorly; heights of posteriormost two maxillary teeth one third the heights of their anterior neighbours; ectopterygoid articulating with the jugal but not with the maxilla.

## Results

### Description

#### Dermal skull roof

**Fig. 1:**
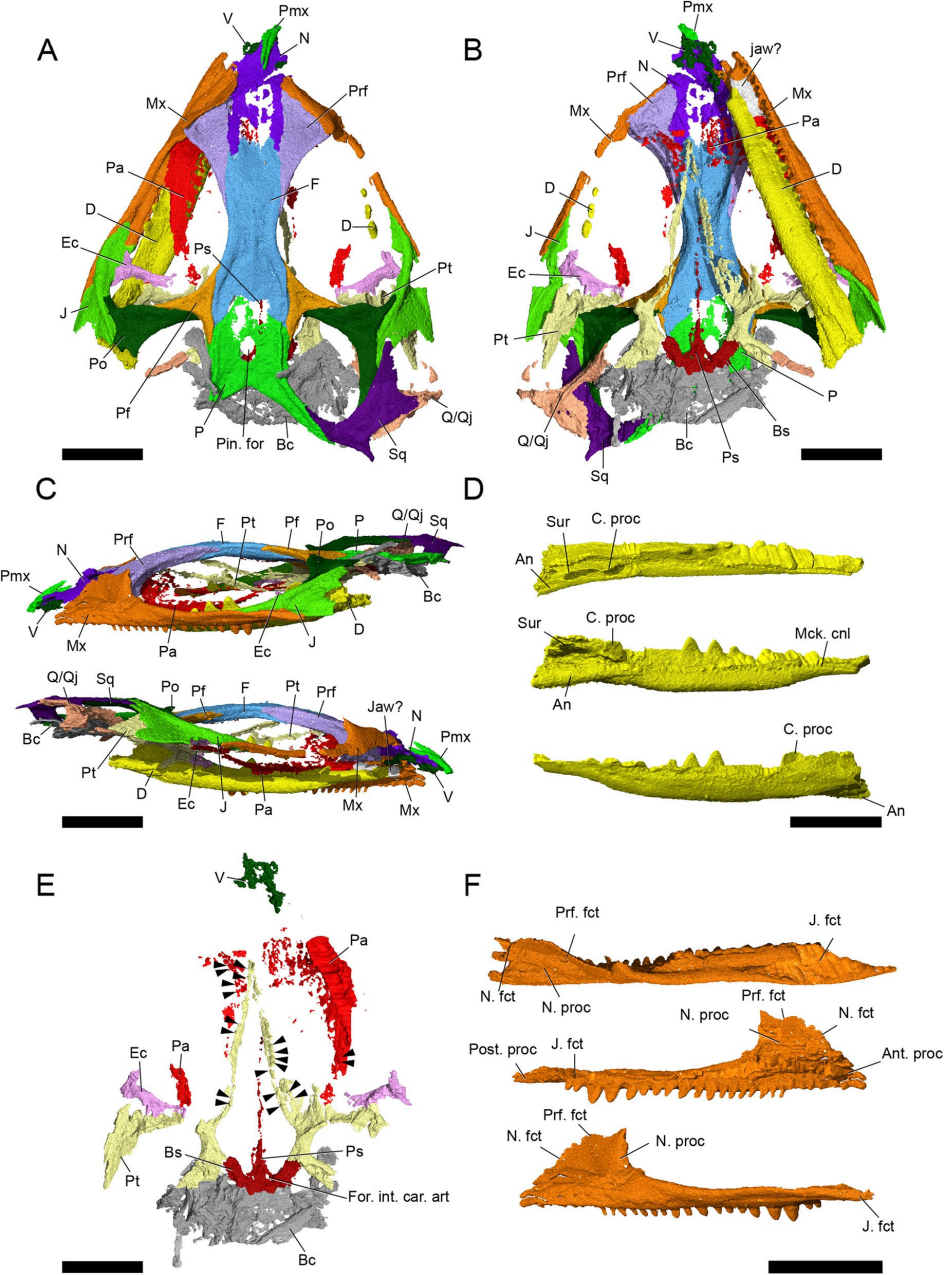
Three-dimensional µCT segmentation of the *Parvosaurus harudensis* holotype specimen MB.R.4520.2. **A** skull in dorsal; **B** in ventral; **C** in left lateral (top), and right lateral (bottom) view; **D** left mandible in dorsal (top), medial (middle), and lateral (bottom) view; **E** palatal bones and braincase in ventral view; **F** left maxilla in dorsal (top), medial (middle), and lateral (bottom) view. Abbreviations: An, angular; Ant. proc, anterior process; Bc, braincase; Bs, basisphenoid; C. proc, coronoid process; D, dentary; Ec, ectopterygoid; F, frontal; For. int. car. art., foramen for internal carotid artery; Jaw?, unidentified fragment, probably jaw fragment; J, jugal; J. fct, jugal facet; Mck. cnl, Meckelian canal; Mx, maxillary; N, nasal; N. fct, nasal facet; N. proc, nasal process; P, parietal; Pa, palatine; Pf, postfrontal; Pin. for, pineal foramen; Pmx, premaxillary; Po, postorbital; Post. proc, posterior process; Prf, prefrontal; Prf. fct, prefrontal facet; Ps, parasphenoid; Pt, pterygoid; Sur, surangular; Sq, squamosal; Q/Qj, quadrate-quadratojugal complex; V, vomer. Arrowheads indicate palatal teeth on the pterygoids and palatines. Scale bar equals 3 mm

With a total length of 16 mm, the skull of *Parvosaurus* *harudensis* is very small in comparison to other sphenodontians, but similar to *Diphydontosaurus avonis* [11]. The specimen is dorsoventrally compressed, but as preserved, the skull is 14 mm wide and 3 mm tall (Fig. 1). In dorsal view, it has a roughly triangular shape and is characterised by very large orbits, spanning over more than one third of the skull length. Its skull roof is mostly preserved, aside from the anterior portions of the nasals, premaxillae, and maxillae, as well as the posterior portions of the left parietal, postorbital, jugal, and squamosal. The left lower jaw and parts of the palate are preserved. Although present, the braincase is not sufficiently well preserved to distinguish between its elements. *Parvosaurus harudensis* has proportionately large orbits and a large upper temporal fenestra positioned dorsolaterally. It is unclear whether the lower temporal bar was fully or partially developed. The parietals are broad and lack a sagittal crest. Large portions of the nasals and parietals are poorly ossified, which suggests that the holotype of *Parvosaurus harudensis* was an immature specimen.

**Fig. 2:**
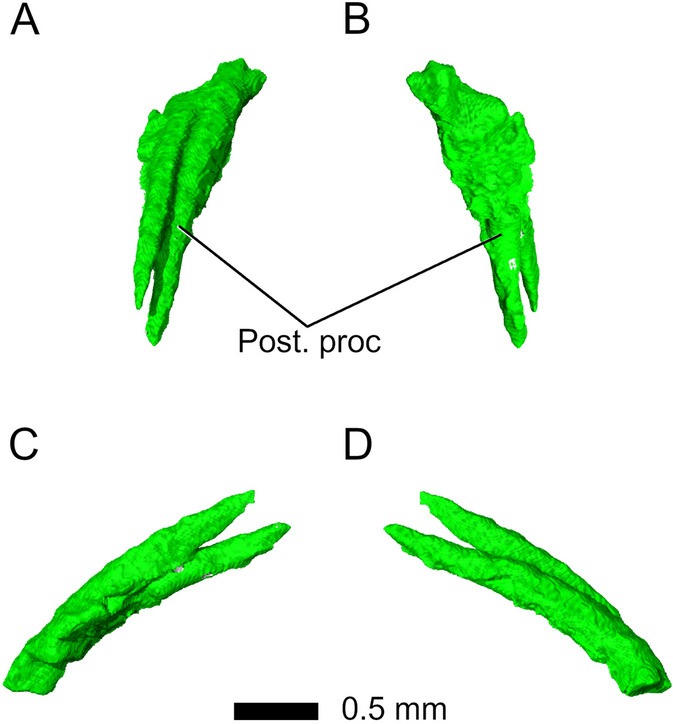
Premaxillae in **A** dorsal, **B** ventral; **C** left lateral, and **D** right lateral view. Abbreviations: Post. proc, posterior process

The *premaxillae* form the anteriormost portion of the skull and are heavily fragmented in *Parvosaurus harudensis*. In dorsal view, they form slender posterodorsal processes that overlap the nasals as in *Gephyrosaurus bridensis* [15] (Fig. 2). It is unclear how far the premaxillae extended posterolaterally to contact the maxillae, and whether they formed the majority of the anterior surface of the snout, as in *Diphydontosaurus avonis* [11]. Neither the position nor the orientation of the external nares can be identified. No tooth-bearing parts of the premaxillae are preserved in *Parvosaurus harudensis.* Viewed from the side, the posterior processes ascend from the tip of the snout over the nasals and end in sharp points (Fig. 2C, D). The right and left processes do not diverge from one another.

**Fig. 3:**
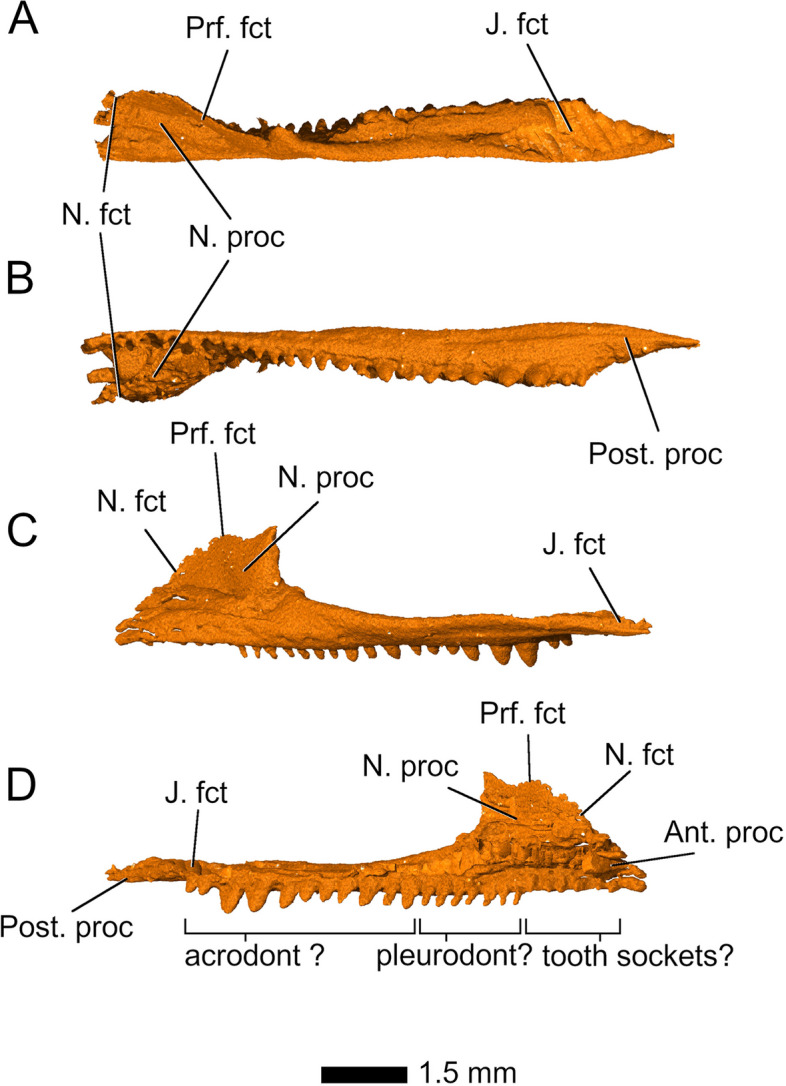
Left maxillary in **A** dorsal; **B** ventral; **C** lateral; and **D** medial view. Abbreviations: Ant. proc, anterior process; J. fct, jugal facet; N. fct, nasal facet; N. proc, nasal process; Post. proc, posterior process; Prf. fct, prefrontal facet

*Parvosaurus harudensis* shows additional similarities to *Diphydontosaurus avonis* in features of the *maxillae*, which are long with a strong overlap of the ventrolateral regions of the prefrontals and a pronounced dorsal process articulating with the nasals (Fig. 3). Most of the left maxilla is preserved (Fig. 3), along with a thin strip of the right posterior maxillary ramus and most of its dorsal process. Anteriorly, both maxillae are strongly fragmented or missing entirely, so that their surfaces articulating with the premaxillae, as well as the maxillary contribution to the external nares, cannot be determined. Their posterior maxillary processes reach two thirds to the level of the ventral orbital margin, contacting the jugals along a posteroventrally sloped surface. Anteriorly on the right maxilla, a series of small lateral foramina are visible. As preserved, the left maxilla bears 18 teeth, whereas only three teeth are preserved on the right side. Since the anterior ends of the maxillae are missing, the total number of teeth cannot be determined with confidence. The anteriormost tooth-bearing section of the left maxilla is most likely missing all teeth, but six shallow bulges are present in that space. We interpret these as tooth 'sockets', rather than shallow teeth. The first 10 preserved teeth increase in size posteriorly, followed by a series of three uniformly smaller teeth and three increasingly larger ones; the last two of these are particularly robust, being four times the height of the anteriormost preserved teeth. The two posteriormost preserved teeth of the maxilla are again very small (Fig. 3C, D). In terms of shape, the anterior, mid, and posterior teeth differ greatly from one another. The ten anteriormost preserved tooth crowns are pin-like with a narrow base and a comparatively broad apex. In contrast, the mid- and posterior teeth are pyramid-shaped and conical with a broad base and sharp apex. No ridges could be identified on any of the tooth crowns, which is possibly attributable to taphonomic erosion.

**Fig. 4:**
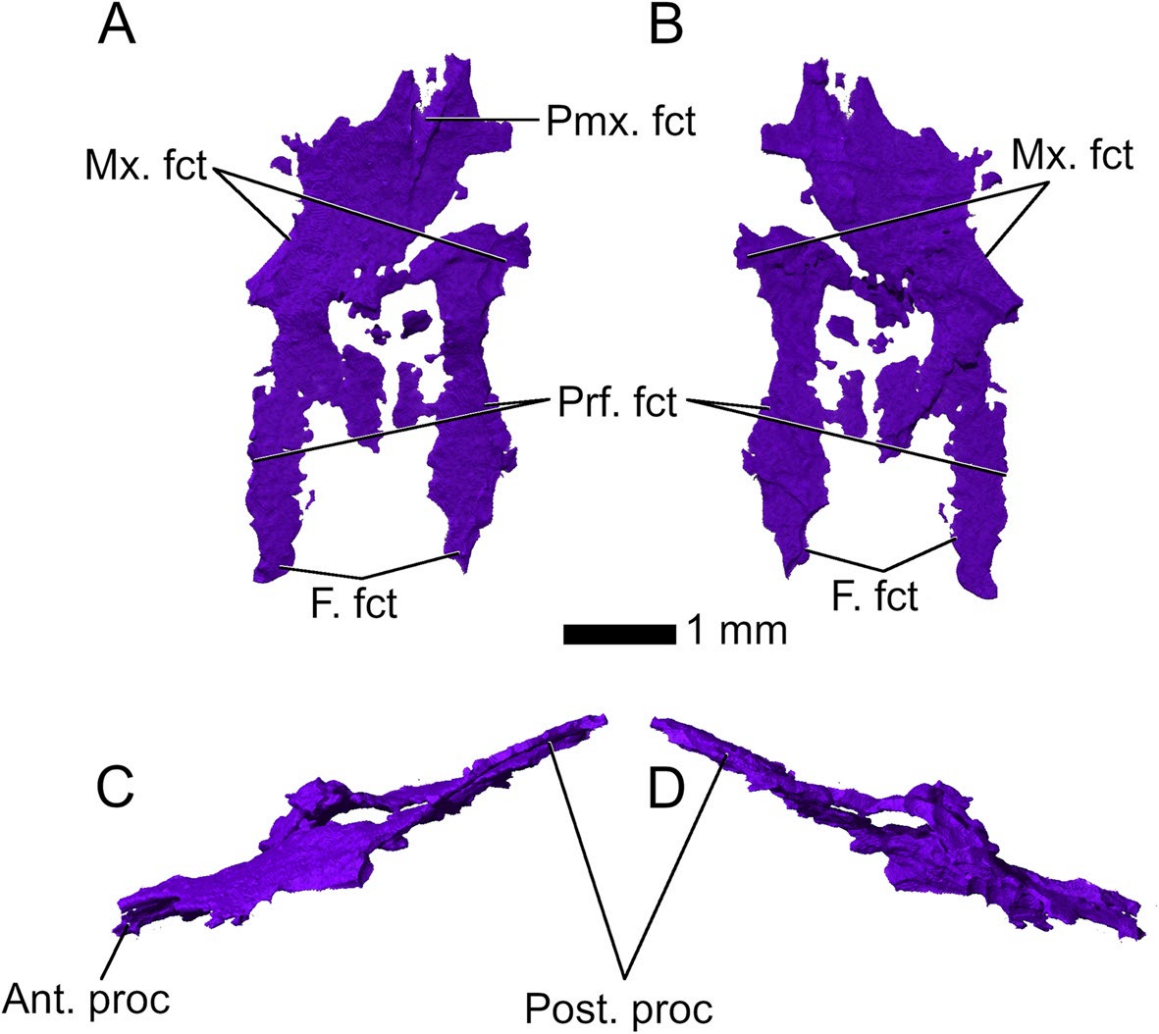
Nasals in **A** dorsal; **B** ventral; **C** left lateral and **D** right lateral view. Abbreviations: Ant. proc, anterior process; F. fct, frontal facet; Mx. fct, maxillary facet; Post. proc, posterior process; Prf. fct, prefrontal facet

The *nasals* are partially preserved with most of their lateral edges present but they are fragmented anteriorly and medially (Fig. 4). They extend over one quarter of the total skull length. Since the medial part is mostly unossified, it is difficult to determine whether the nasals were fused or paired elements. They are equally wide anteriorly and posteriorly (Fig. 4), in contrast to other basal rhynchocephalians including *Diphydontosaurus avonis* [11] and *Gephyrosaurus bridensis* [15], which share anteriorly broad and posteriorly narrowing nasals, as well as *Planocephalosaurus robinsonae* [29], in which the nasals are broad at mid-length and become narrower anteriorly and posteriorly. The lateral margins extend straight anteroposteriorly with very broad contact surfaces for the maxillae and prefrontals, ending posteriorly in a transverse sutural contact with the frontals. Exceptions to this are two posterolateral processes, which extend posteriorly along the lateral edges of the frontals.

**Fig. 5:**
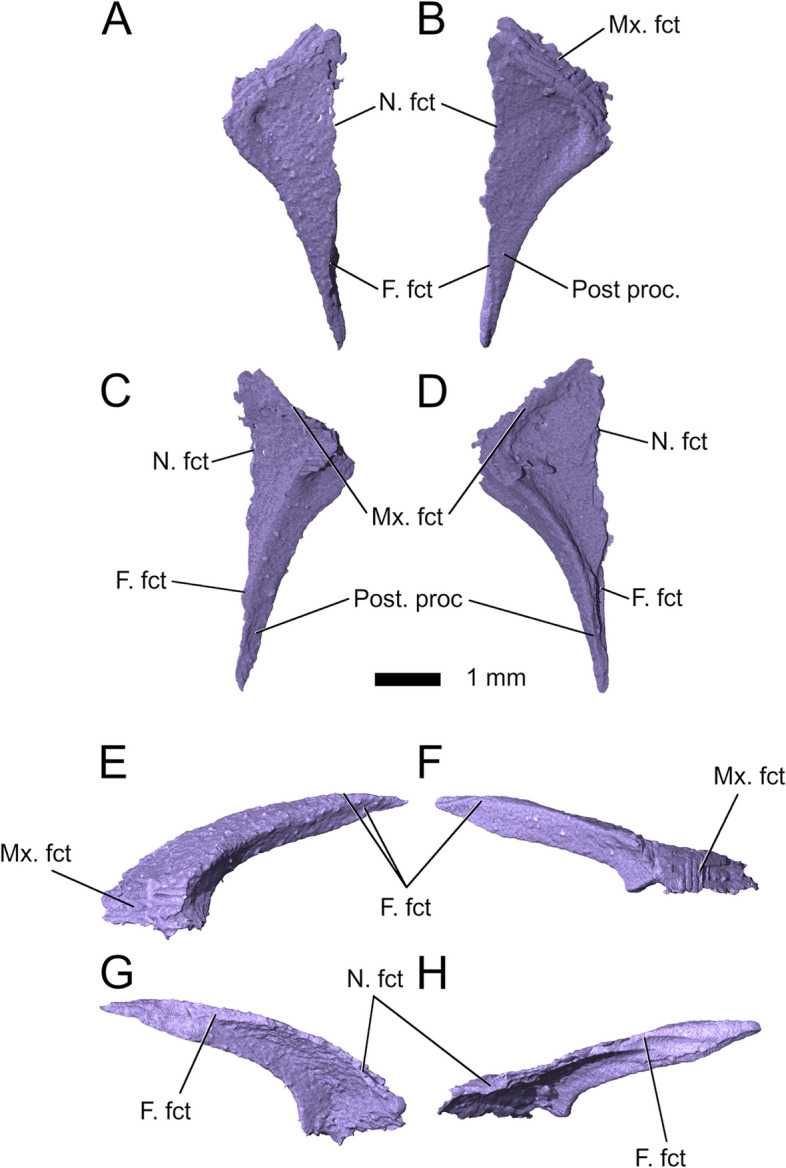
Right prefrontal in **A** dorsal; **C** ventral; **E** lateral; and **G** medial view. Left prefrontal in **B** dorsal; **D** ventral; **F** lateral; and **H** medial view. Abbreviations: F. fct, frontal facet; Mx. fct, maxillary facet; N, fct, nasal facet; Post. proc, posterior process

In dorsal view, the *prefrontals* are triangular in outline with a broad anterolateral contact with the maxilla (Fig. 5). On the posterior half of their posteromedial edge, they bear broad facets for the frontals. They become significantly more slender in the posterior region and terminate in a narrow tip along the lateral edges of the frontal posteriorly. On the anterolateral edge, the prefrontals bear broad facets for the maxillae and facets for the frontal along the posterior half of their posteromedial margins. Sharp ridges on their ventral surface mark the limits of the nasal chamber and continue posteriorly as similarly sharp ridges on the frontals.

*Lacrimals* are absent in *Parvosaurus harudensis*. This is also the case in some lepidosaurs, the lepidosauromorph *Taytalura alcoberi* [30], and the plesiomorphic condition for rhynchocephalians [31].

**Fig. 6:**
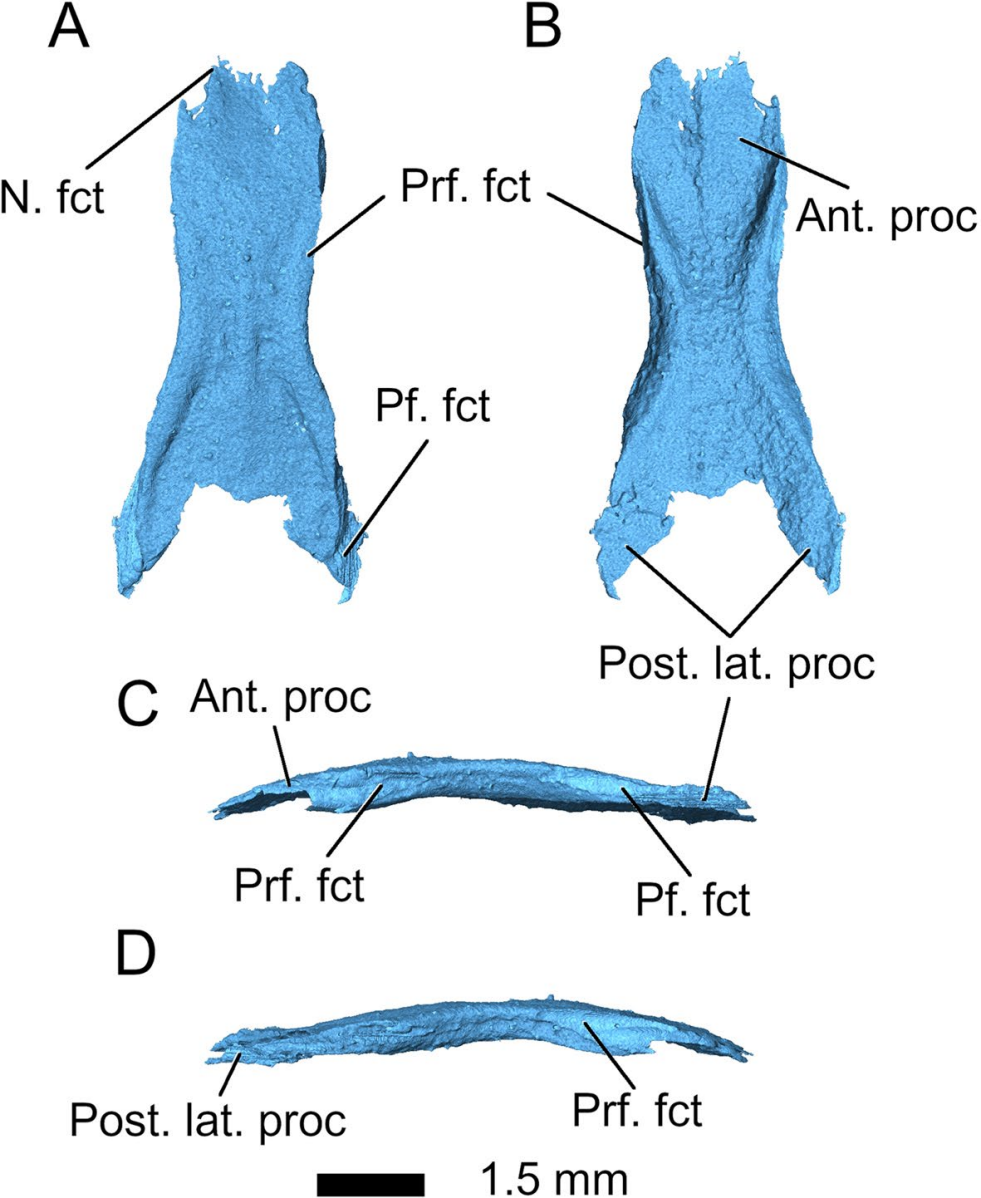
Frontal in **A** dorsal; **B** ventral; **C** left lateral; and **D** right lateral view. Abbreviations: Ant. Proc, anterior process; N. fct, nasal facet; Pf. Fct, postfrontal facet; Post. lat. Proc, posterolateral process; Prf. fct, prefrontal facet

The *frontals* of *Parvosaurus harudensis* are fused in a single, thick element, forming the supraorbital margin for a short distance between the prefrontals and postfrontals. Their dorsal surface is smooth without any noticeable ornamentation. The sutural lines are easily distinguishable on the posterior end, separating the frontal from the postfrontals and parietal. The anterior sutures with the nasals and prefrontals are less visible. A shallow ridge extends anteroposteriorly through the centre of the compound frontal bone, marking the original separation between left and right elements. In dorsal view, the frontal closely resembles those of *Clevosaurus hudsoni *[32], *Planocephalosaurus robinsonae *[29]*,* and *Diphydontosaurus avonis* [11] in the presence of posterolaterally oriented processes, which form part of the sutures with the parietals. They do not narrow anteriorly as strongly as in *Diphydontosaurus avonis* [11] and *Gephyrosaurus bridensis* [15], where the naso-frontal suture becomes narrower anteriorly with the posterolateral processes of the nasals extending alongside it. Instead, the naso-frontal suture of *Parvosaurus harudensis* is aligned more transversely (Fig. 1A). The width of the frontals remains unchanged from its centre to the naso-frontal suture (Fig. 6), much as in *Planocephalosaurus robinsonae* [29] and *Clevosaurus hudsoni* [32], which also share its slight broadening of the posterior region. Additionally, the presence of posterolaterally oriented processes, which form part of the sutures with the parietals, also closely resembles the condition in *Clevosaurus hudsoni* [32], *Planocephalosaurus robinsonae* [29] and *Diphydontosaurus* [11].

**Fig. 7:**
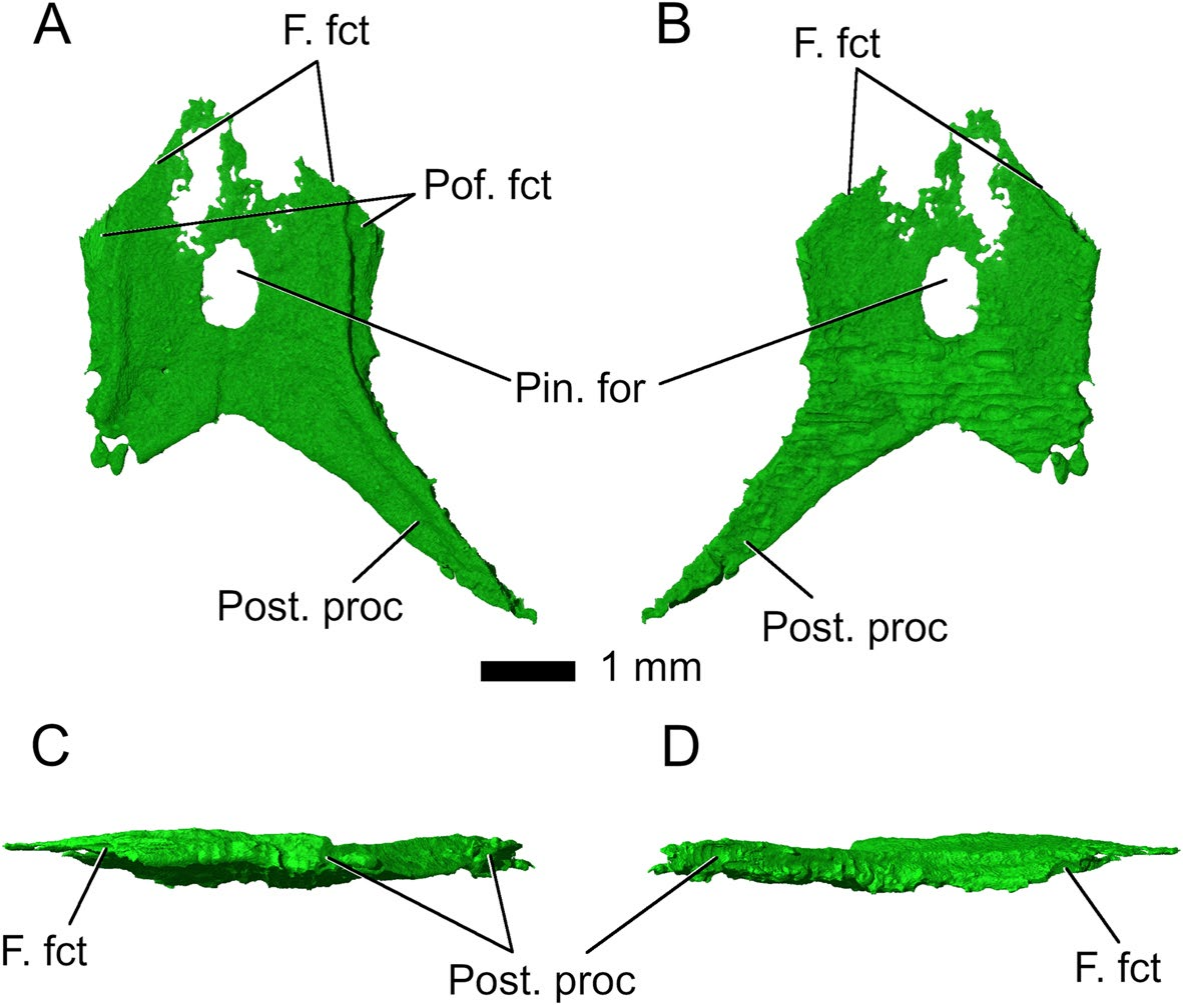
Parietal in **A** dorsal; **B** ventral; **C** left lateral; and **D** right lateral view. Abbreviations: F. fct, frontal facet; Pin. for, pineal foramen; Post. proc, posterior process

The *parietals* in *Parvosaurus harudensis* are fused and surround a slightly elongated, well-developed pineal foramen. They are generally broad and lack long posterior processes as well as a sagittal crest (Fig. 7). The parietals are poorly ossified anteriorly. The contact with the frontal proceeds transversely but with a slight anteriorly convex curve. They do not contact the postorbitals and form only very short contacts with the postfrontals. At the posterior end, the posterior processes contact the squamosals. In dorsal view, the body of the parietals is narrow and generally similar to *Clevosaurus hudsoni* [32] and *Gephyrosaurus bridensis* [15]. The posterior processes in *Gephyrosaurus bridensis*, like those of *Parvosaurus harudensis*, include a 110° angle between them, with an anteriorly convex emargination forming the posterior margin of the parietals. In contrast, the postparietal processes in *Clevosaurus bridensis* [32] and *Planocephalosaurus robinsonae* [29] form a broad, straight posterior margin rather than an embayment [33].

**Fig. 8:**
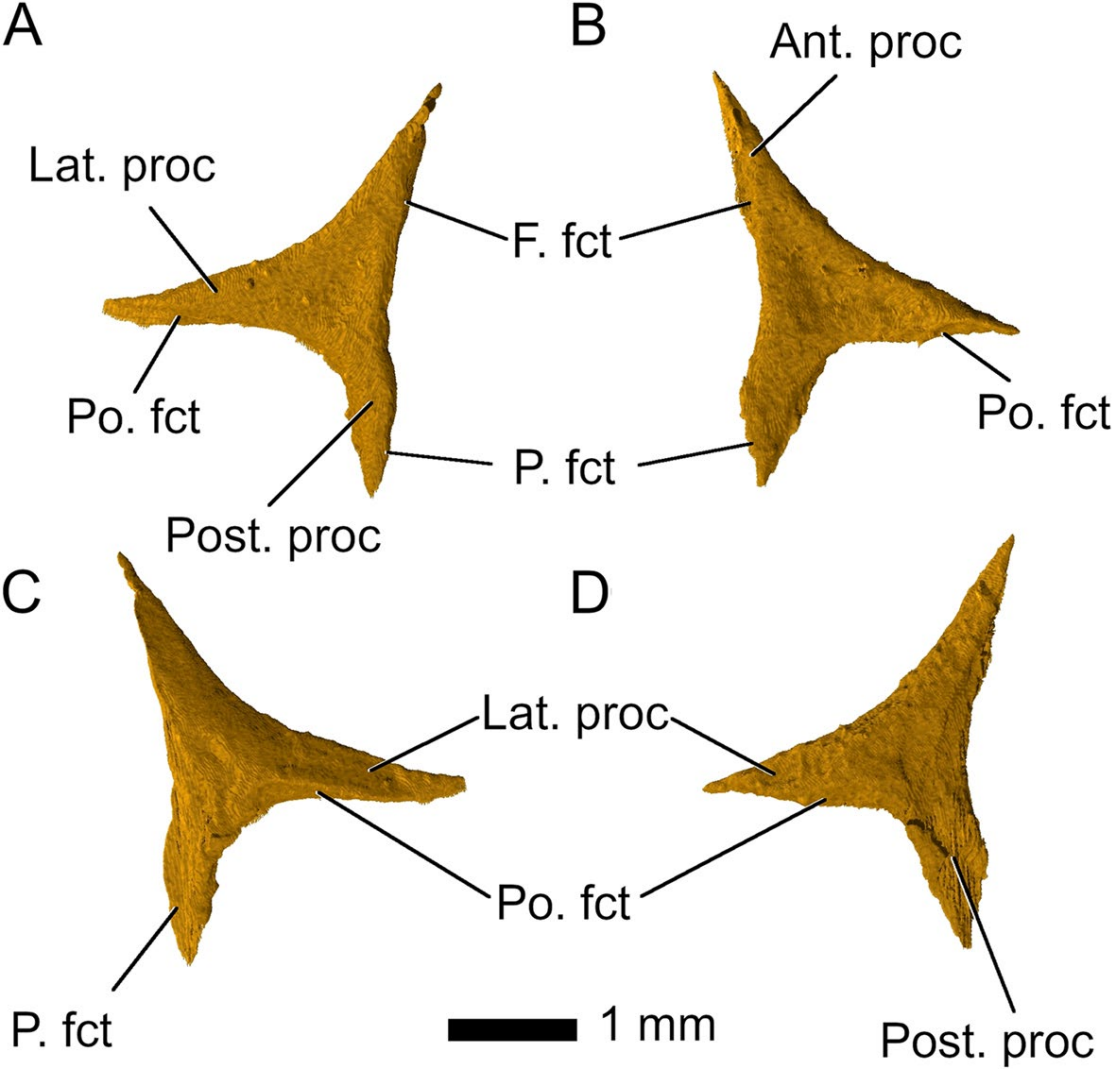
Left postfrontal in **A** dorsal; and **C** ventral view. Right postfrontal in **B** dorsal; and **D** ventral view. Abbreviations: Ant. Proc, anterior process; F. fct, frontal facet; Lat. Proc, lateral process; P. fct, parietal facet; Po. Fct, posterior facet; Post. proc, posterior process

The *postfrontals* of *Parvosaurus harudensis* are triradiate and overlap the postorbitals with their ventrolateral processes (Fig. 8), unlike in *Planocephalosaurus robinsonae* [29], where the ventrolateral processes underlap the postorbitals. Their medial edges contact the frontal except for the very tip of their posterior processes, which contact the anterior ends of the parietals. The anterior processes articulate with the lateral edges of the frontal. In comparison, the ventrolateral process in *Diphydontosaurus avonis* is almost entirely covered by the postorbital [11]. The anterior processes form the posterodorsal corners of the orbital margins. In *Parvosaurus harudensis*, the postfrontals extend over the posterior limits of the frontals, whereas in *Diphydontosaurus avonis* [11], they end posteriorly at the frontoparietal suture. Moreover, as in *Diphydontosaurus avonis*, the ventral processes are noticeably longer than the anterior ones [11] while they are of approximately equal length in *Parvosaurus harudensis*.

**Fig. 9:**
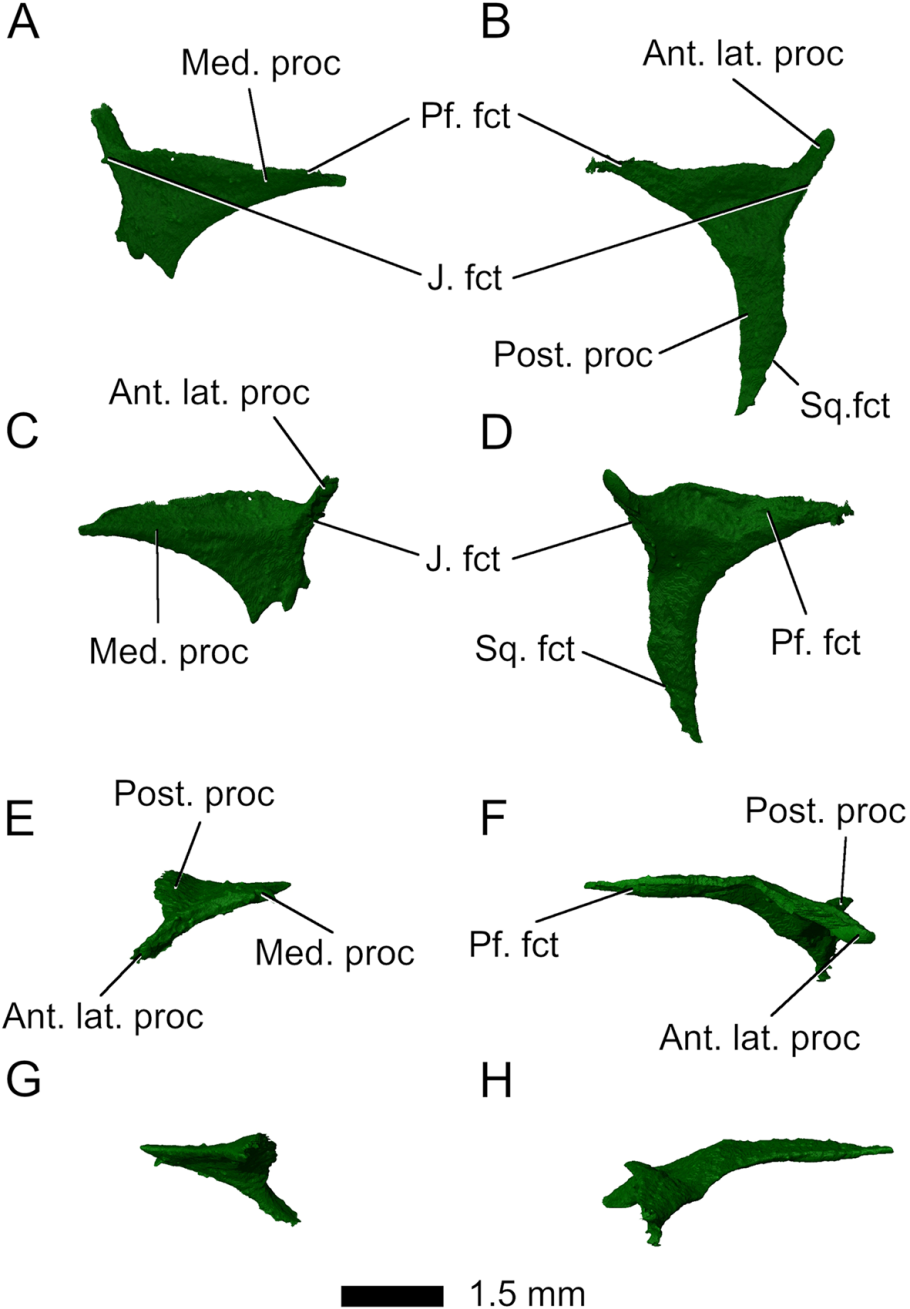
Left (**A**, **C**, **E**, **G**) and right (**B**, **D**, **F**, **H**) postorbital in **A**, **B** dorsal; **C**, **D** ventral; **E**, **F** left lateral; **G**, **H** right lateral view. Abbreviations: Ant. lat. proc, anterolateral process; J. fct, jugal facet; Med. proc, medial process; Pf. fct, postfrontal facet; Post. proc, posterior process; Sq. fct, squamosal facet

*Parvosaurus harudensis* has large *postorbitals* (Fig. 9). The left postorbital is partially preserved (Fig. 9A, C, E) whereas the right one is complete (Fig. 9B, D, F). The postorbitals contact the postfrontals dorsally and form the posterior margins of the orbits. They are similar in shape to the postfrontals, generally triangular and T-shaped. The long posterior processes articulate with the squamosals, the much shorter ventral processes attach to the jugals. The posterior processes form more than half of the lateral rims of the supratemporal fenestrae and are excluded from the infratemporal fenestrae by the contacts between the jugals and squamosals (Fig. 1C), as is common in many rhynchocephalians including *Gephyrosaurus bridensis* [15], *Diphydontosaurus avonis* [11], and *Planocephalosaurus robinsonae* [29] (Supplementary Fig. S1).

**Fig. 10:**
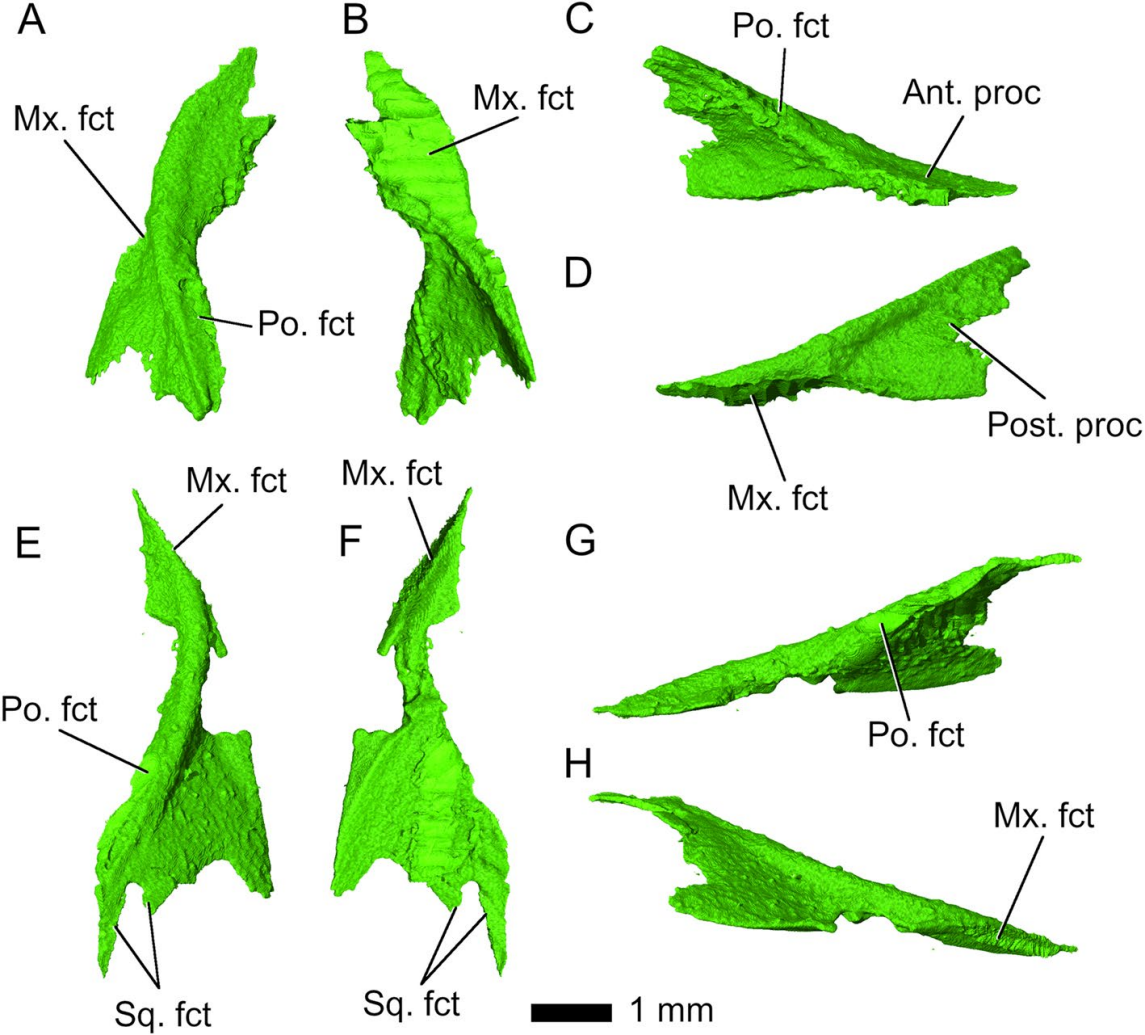
Left (**A**-**D**), and right (**E**-**H**) jugals in **A**, E dorsal; **B**, F ventral; **C**, G medial and **D**, H lateral view. Abbreviations: Ant. Proc, anterior process; Mx. fct, maxillary facet; Po. fct, postorbital facet; Post. proc, posterior process; Sq. fct, squamosal facet

Both *jugals* are incomplete in *Parvosaurus harudensis*. The posterior processes of the jugals, forming the lower temporal arcades, are fragmentary (Fig. 10), but extend at least beyond the midpoint of the infratemporal fenestrae posteriorly, forming a partial or possibly complete infratemporal bar [34]. Their anterior processes articulate diagonally with the maxillae and extend along them to about the midpoints of the orbits. The jugals bear facets for the ectopterygoids and form the posteroventral parts of the orbital margins. Medially, they overlap with the postorbitals, almost covering their entire lateral margins.

**Fig. 11:**
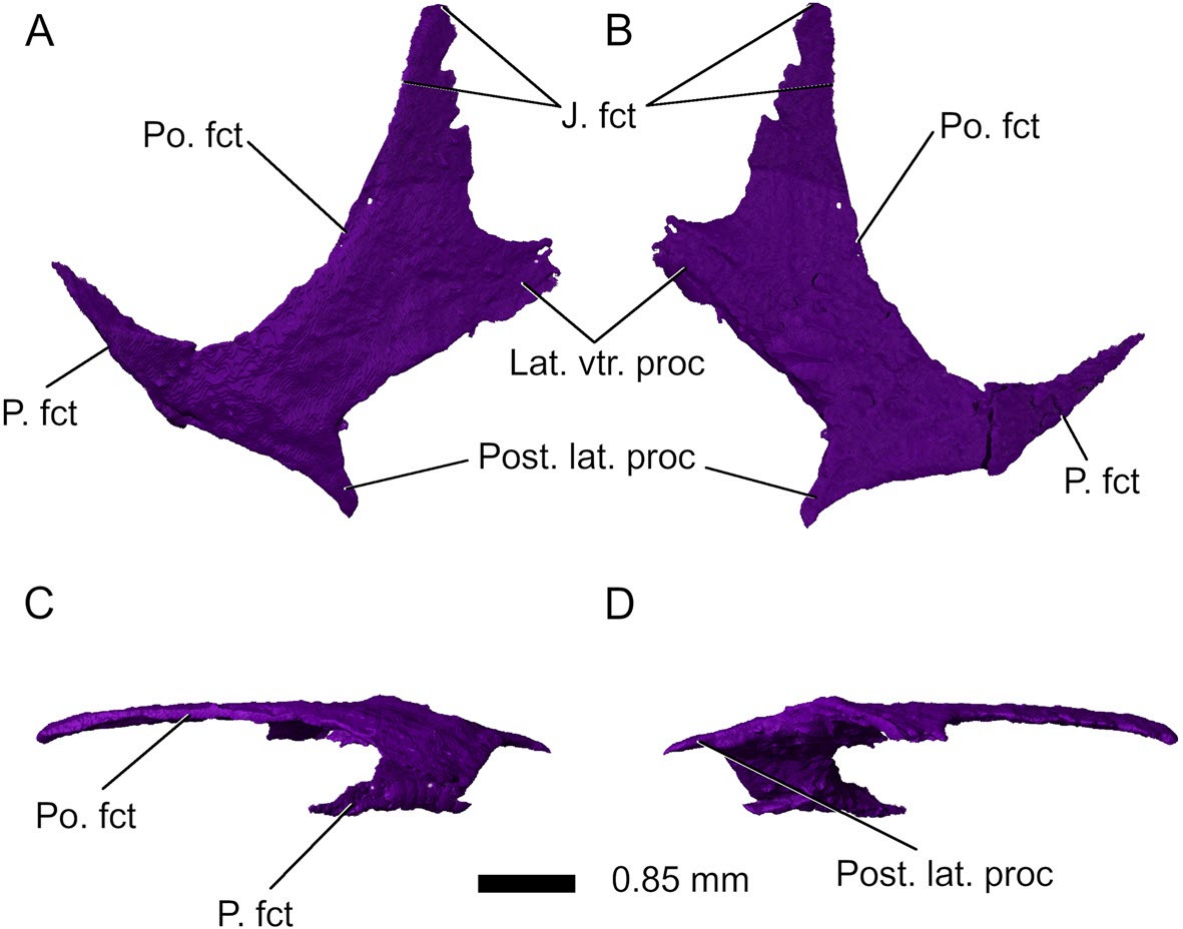
Right squamosal in **A** dorsal, **B** ventral, **C** medial, and **D** lateral view. Abbreviations: J. fct, jugal facet; Lat. vtr. proc, lateroventral process; P. fct, parietal facet; Po. fct, postorbital facet; Post. lat. proc, posterolateral process

Most of the right *squamosal* has been preserved (Fig. 11), but the left squamosal is missing. The squamosals in *Parvosaurus harudensis* are broad and their anterior processes are ventrally directed (Fig. 11), articulating with both the postorbital and the jugal, whereas the dorsal processes contact the parietals diagonally. They strongly overlap the quadrate-quadratojugal complex. The main body of the squamosal forms the posterior margin of the supratemporal fenestra as well as the posterodorsal margin of the infratemporal fenestra. There is a short, tapered posterior process. The suture with the posterior process of the parietal is clearly visible.

Supratemporals were not found and, as in *Planocephalosaurus robinsonae* [29] and *Diphydontosaurus avonis* [11], they were probably absent since there are no facets on the postparietal processes that would suggest their presence (Figs. 1A and 11). A small triangular piece of bone articulating posterolaterally with the left posterior process of the parietal is situated in the appropriate position. However, its contact with the squamosal as well as the general shape of the fragment strongly suggest that the contact is a break within the squamosal rather than a suture between multiple bones. Therefore, the fragment is considered part of the squamosal rather than a supratemporal.

**Fig. 12:**
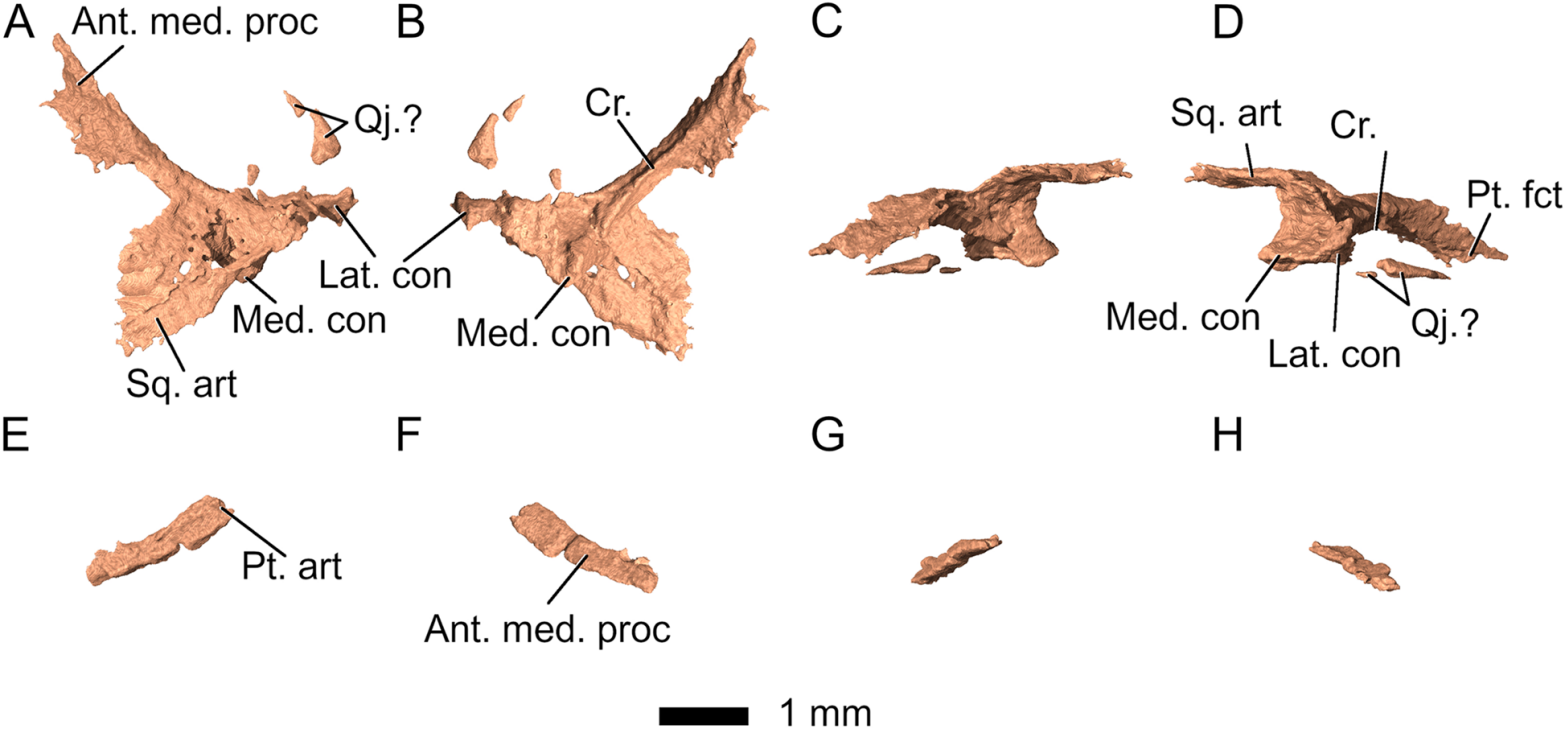
Right (**A**-**D**) and left (**E**-**H**) quadrate-quadratojugal complex in **A**, E dorsal; **B**, F ventral; **C**, G medial; **D**, H lateral view. Abbreviations: Ant. med. proc, anteromedial process; Cr, crest; Lat. con, lateral condyle; med. con, medial condyle; Pt. fct, pterygoid facet; Sq. art, squamosal articulation; Qj.?, possibly quadratojugal fragments

The *quadrate-quadratojugal* complex forms the junction between the dermal skull roof, the palate, and the mandible and is situated at the posterior end of the cranium, extending ventrally to meet the pterygoids. In *Parvosaurus harudensis*, it is only partially preserved on both sides of the cranium, mostly on the right side (Fig. 12). The quadrate and quadratojugal are conjoined (Fig. 12). However, their fragmentary state does not allow for detailed description of the shape and structure of the quadratojugal. The left quadrate is preserved only as a small, slender fragment articulating with the pterygoid. The suture separating the pterygoid and the quadrate is barely visible. On the right side, it forms a somewhat triradiate shape in which an elongated medial flange articulates with the quadrate flange of the pterygoid, whereas a much wider lateral flange widely underlaps the squamosal (Fig. 1B, C). Small separate bone fragments probably belonging to the quadrate-quadratojugal complex extend this lateral process anteriorly, forming an at least partially complete infratemporal bar (Figs. 1C and 12). However, due to fragmentation, the extent of the quadratojugal contribution to the lower temporal arcade in *Parvosaurus harudensis* cannot be assessed. The quadrate-quadratojugal complex extends anteromedially through a long process, meeting the posterolateral process of the pterygoid. However, the pterygoid facet is not preserved in the right-hand quadrate-quadratojugal fragment. Ventrally, the quadrate forms a double condyle much like in *Diphydontosaurus avonis* [11]. The larger medial condyle is preserved, although the posterolateral portion of the quadrate-quadratojugal complex is fragmented. A posterolateral crest extends from the point of articulation with the pterygoid to the medial condyle. The lateral condyle is only partially preserved, providing little information on its articular surface.

#### Palate

**Fig. 13:**
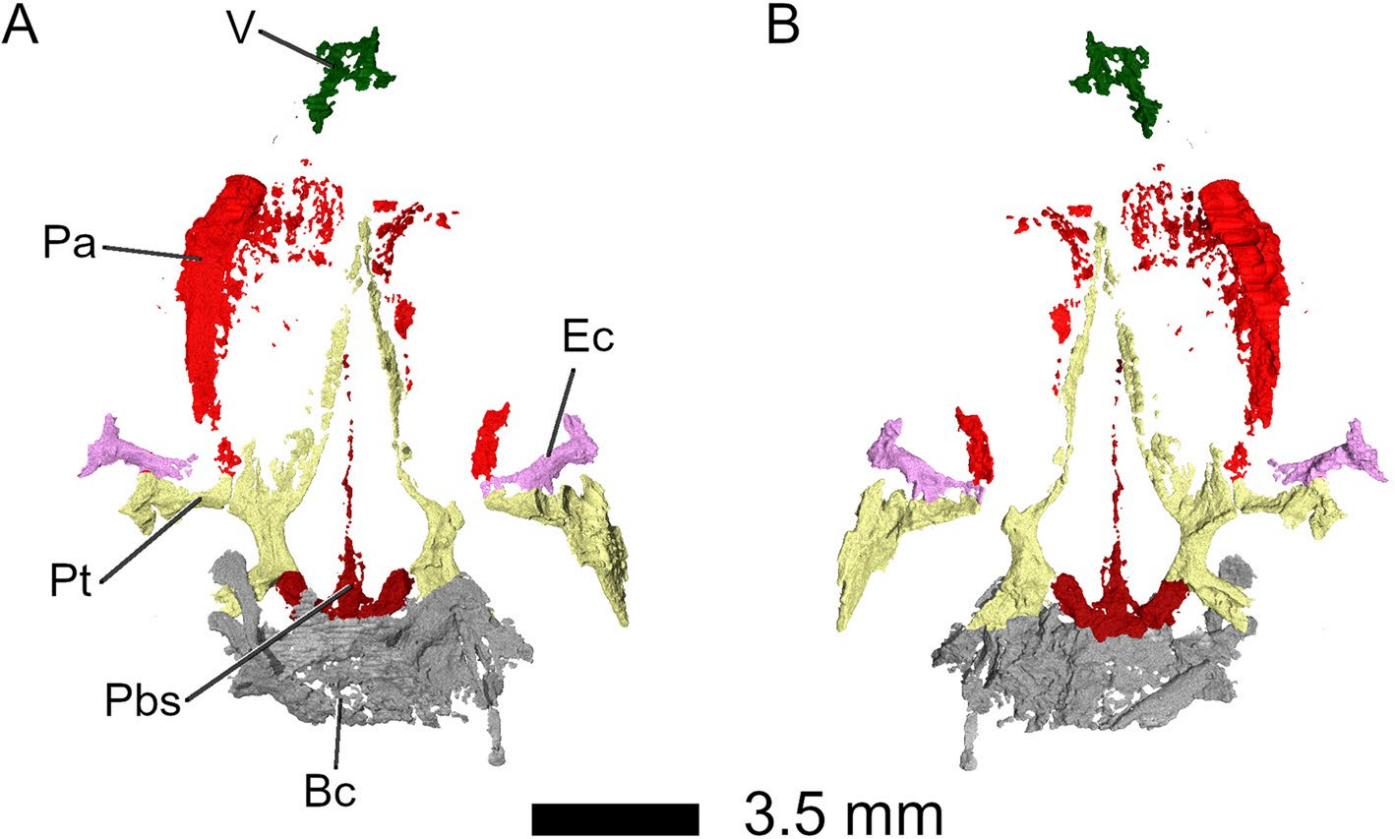
Palate in **A** dorsal, and **B** ventral view. Abbreviations: Bc, braincase; Ec, ectopterygoid; Pa, palatine; Pbs, parabasisphenoid; Pt, pterygoid

**Fig. 14:**
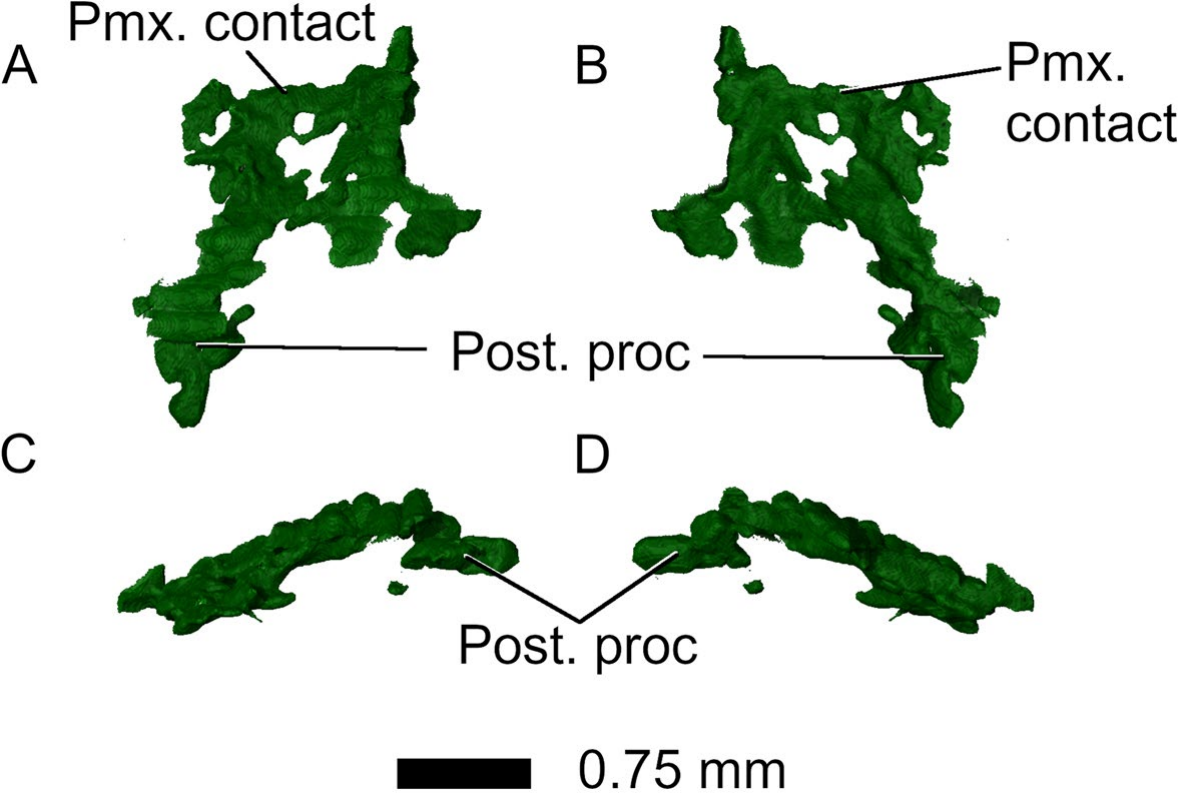
Vomer in **A** dorsal, **B** ventral, **C** left lateral and **D** right lateral view. Abbreviations: Pmx. contact, premaxillary contact; Post. proc, posterior process

Parts of the palate are poorly preserved, but many structures can still be identified (Figs. 1E and 13). The *vomers* in *Parvosaurus harudensis* are paired, mostly triangular in shape, and form the anterior part of the palate (Fig. 14). Only small fragments of the anterior region are preserved. Due to the preservational state of the vomers, surfaces articulating with neighbouring bones including the pterygoids cannot be clearly identified. The preserved portions of the vomers extend from the posterior processes of the premaxillae to the centre of the nasals. Generally, rhynchocephalians possess fully toothed palates [18], but the vomers in *Parvosaurus harudensis* bear no visible teeth (Fig. 14). The position of the choana could not be determined due to the poor preservation.

**Fig. 15:**
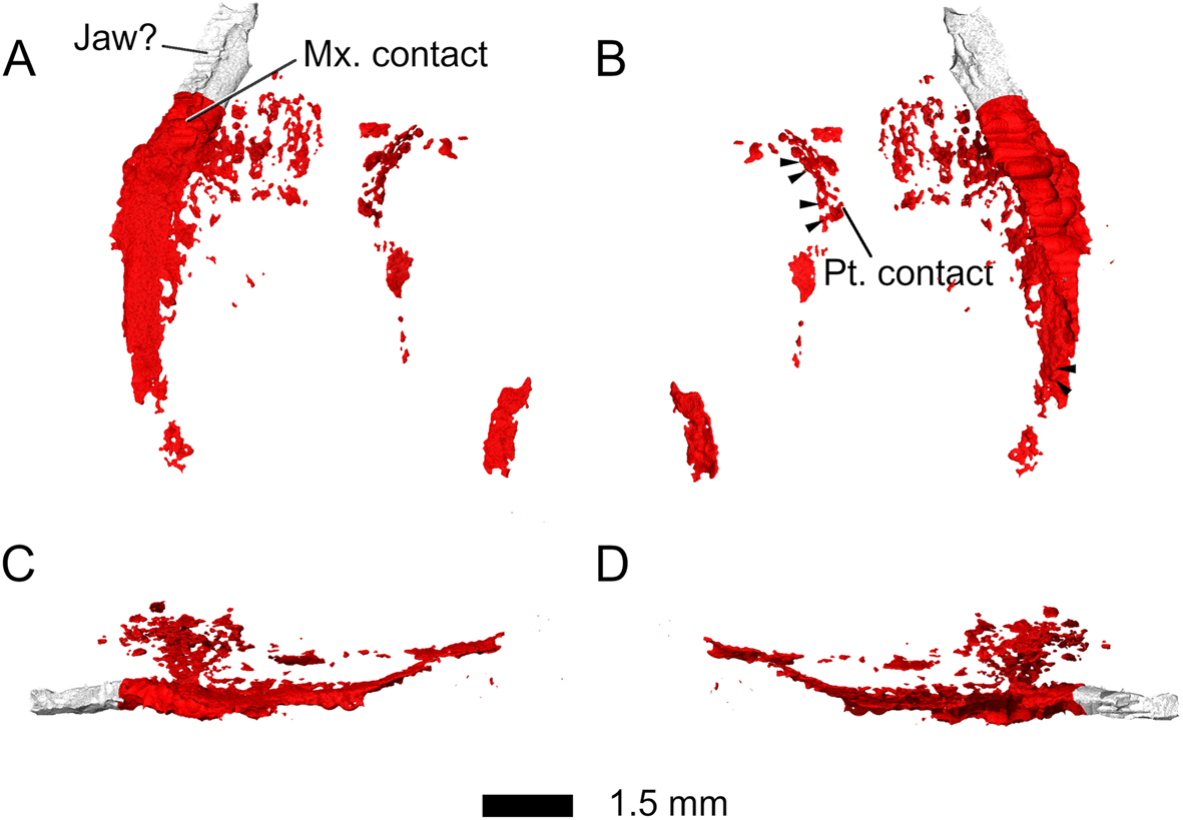
Palatines in **A** dorsal, **B** ventral, **C** left lateral, and **D** right lateral view. Abbreviations: Jaw?, unknown fragment, possibly jaw; Mx. contact, maxillary contact; Pt. contact, pterygoid contact

**Fig. 16:**
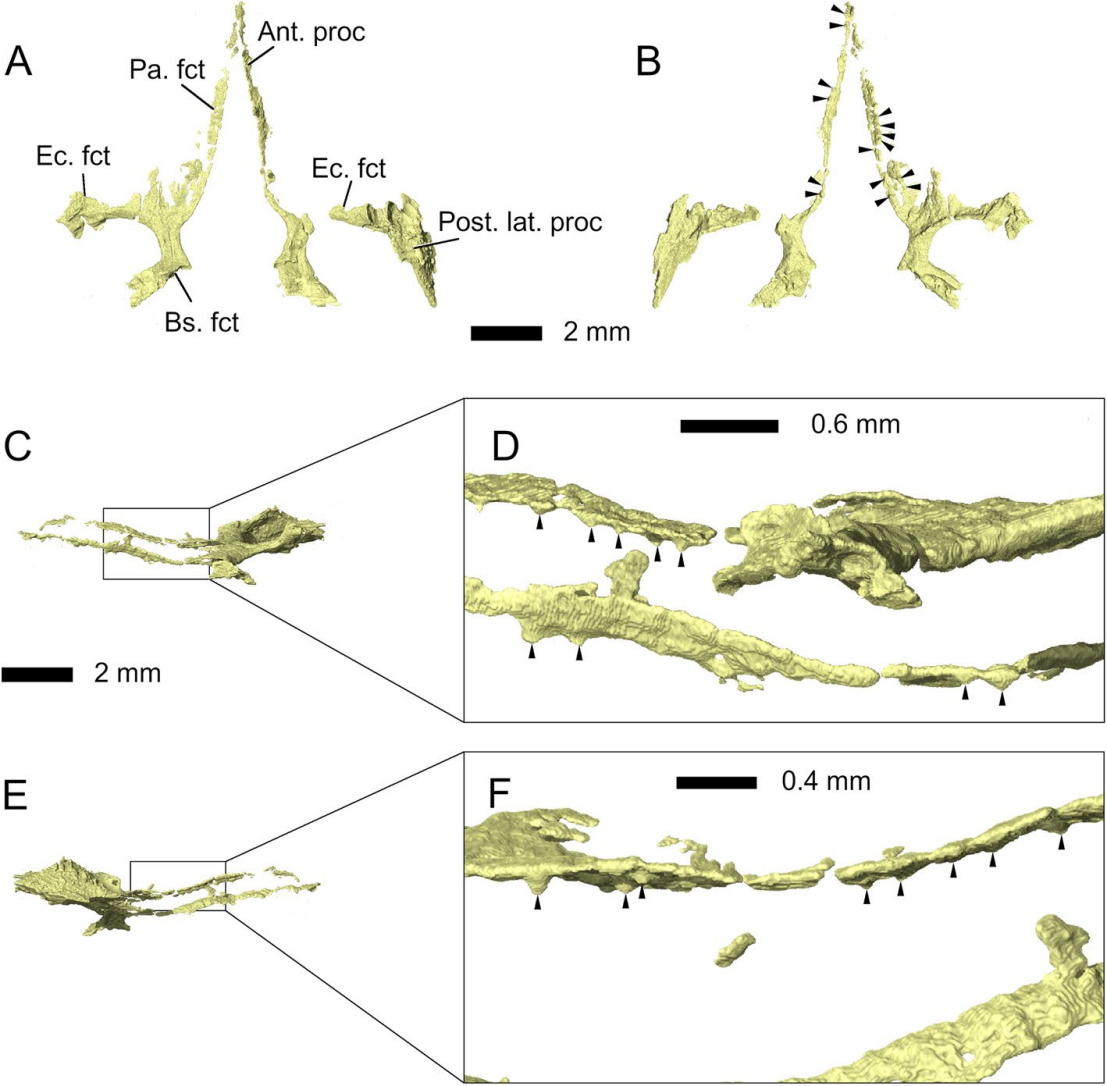
Pterygoids in **A** dorsal, **B** ventral, **C**-**D** left lateral, and **E**-**F** right lateral view. Abbreviations: Ant. proc, anterior process; Bs. fct, basisphenoid facet; Ec. fct, ectopterygoid facet; Pa. fct, palatine facet; Post. lat. proc, posterolateral process. Arrowheads indicate the position of palatal teeth on the pterygoids

The *palatines* form the medial part of the palate and occupy the largest area of all the palatal bones, along with the pterygoids (Fig. 15). The palatines are highly fragmented but apparently make broad contact with the maxillae (Fig. 15). However, their fragmentary state does not allow identification of the position of the nares. Several small palatal teeth could be identified on the posterior region of the left palatine (Fig. 1E), but the number as well as orientation of possible tooth rows cannot be assessed. Small palatal teeth could also be identified in the lateral region of the pterygoids, but as in the palatines, no distinct tooth rows could be determined (Figs. 1E and 16). It is likely that the palate had an extensive tooth cover similar to those in other rhynchocephalians [18].

The *pterygoids* are long bones extending from the anterior edges of the frontals to the midpoints of the supratemporal fenestrae. In *Parvosaurus harudensis*, they are partially preserved on both sides of the palate (Fig. 16). In ventral view, both pterygoids meet at the level of the fronto-nasal suture and form a long and broad interpterygoid vacuity, framing the long cultriform process of the parabasisphenoid. They likely contacted the palatines along the entire lateral margins of the anterior process. However, the articulating surfaces are not preserved in *Parvosaurus harudensis*. On the lateral parts of their ventral surfaces, 15 teeth could be identified on the pterygoids (Figs. 1E and 16B, D, F). However, not much information on tooth rows can be deducted from the scan due to the fragmentary condition of the pterygoids. Posteriorly, the interpterygoid vacuity becomes wider and reaches its widest point at the level of the fronto-parietal suture. The pterygoids form strong transverse flanges that extend to meet the posterior tips of the ectopterygoids, much as in *Gephyrosaurus bridensis* [15] and *Diphydontosaurus avonis* [11]. However, they extend much farther posteriorly along the lateral borders of the suborbital fenestrae before ending at the level of the contacts between pterygoid and quadrate. This is similar to the condition in *Brachyrhinodon taylori* [9] and other clevosaurids [35]. Posteriorly, the pterygoids articulate with the basipterygoid processes and curve laterally from there to meet the quadrates.

**Fig. 17:**
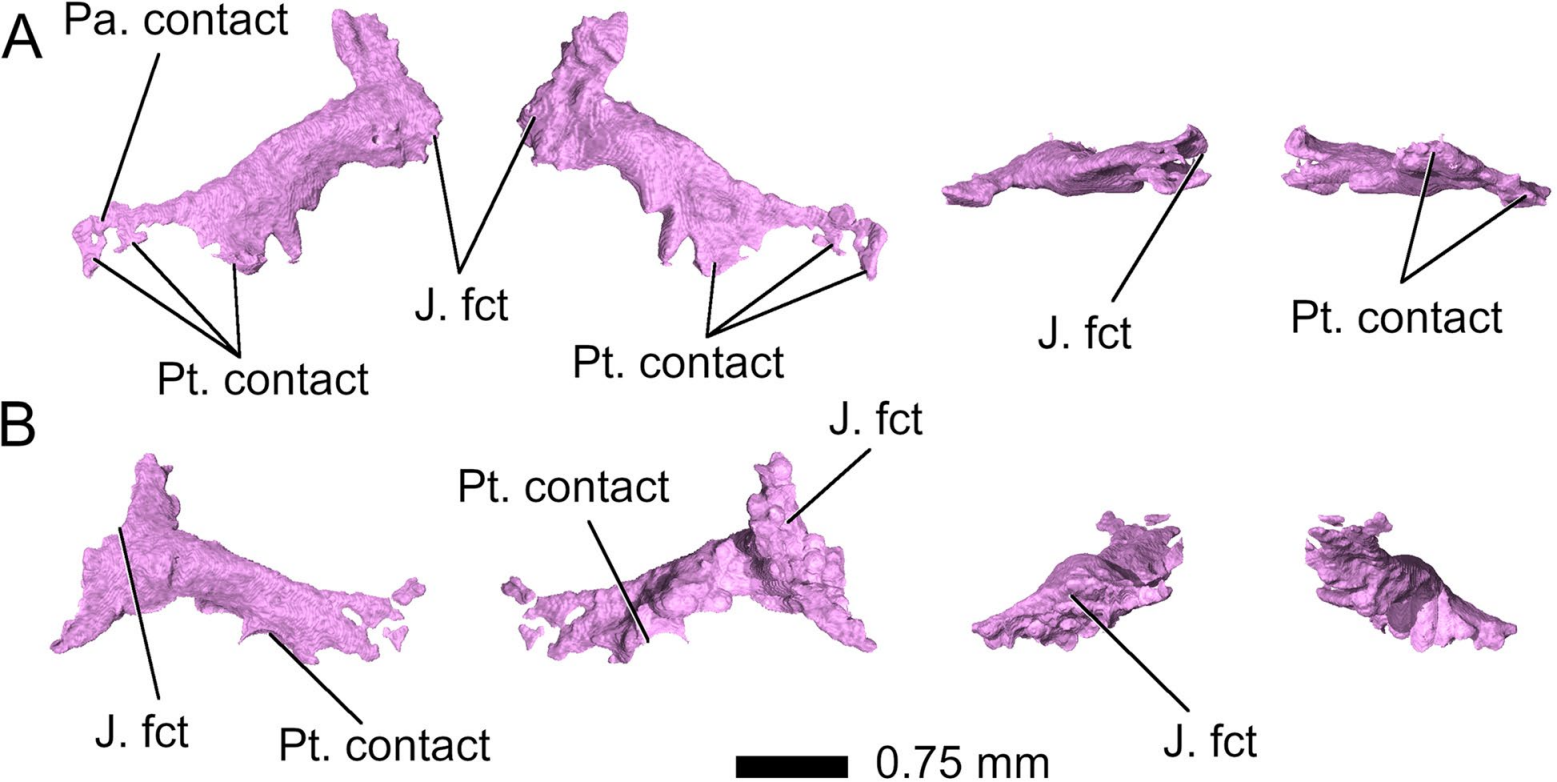
**A** right and **B** left ectopterygoid. Left to right: dorsal, ventral, medial, and lateral view. Abbreviations: J. fct, jugal facet; Pa. contact, palatine contact; Pt. contact, pterygoid contact

The *ectopterygoids* are bar-shaped bones bracing the palate between the pterygoids, jugals, and maxillae. In *Parvosaurus harudensis*, they are partially preserved on both sides of the palate (Fig. 17). They are composed of expanded distal and proximal ends linked by a cylindrical rod. Unlike in *Diphydontosaurus avonis* [11], the distal ends are extended anteriorly along their contacts with the upper jaws in *Parvosaurus harudensis*. The spoon-shaped proximal ends are fragmented on both sides. Additionally, the articular contact with the jugal is not well preserved in the right ectopterygoid (Fig. 17A). Based on the left side of the cranium it can be inferred that the ectopterygoids broadly articulated with the jugals and extended medially to meet the pterygoids with which they have an extensive contact posteriorly. As in *Diphydontosaurus avonis* [11], the ectopterygoids in *Parvosaurus harudensis* articulate with the jugals, but not with the maxillae (Figs. 1E and 17). It is difficult to determine the suture between the ectopterygoids and the pterygoids because both bones are quite fragmented, but there apparently was a large area of contact along the posterior edges of the bones.

**Fig. 18:**
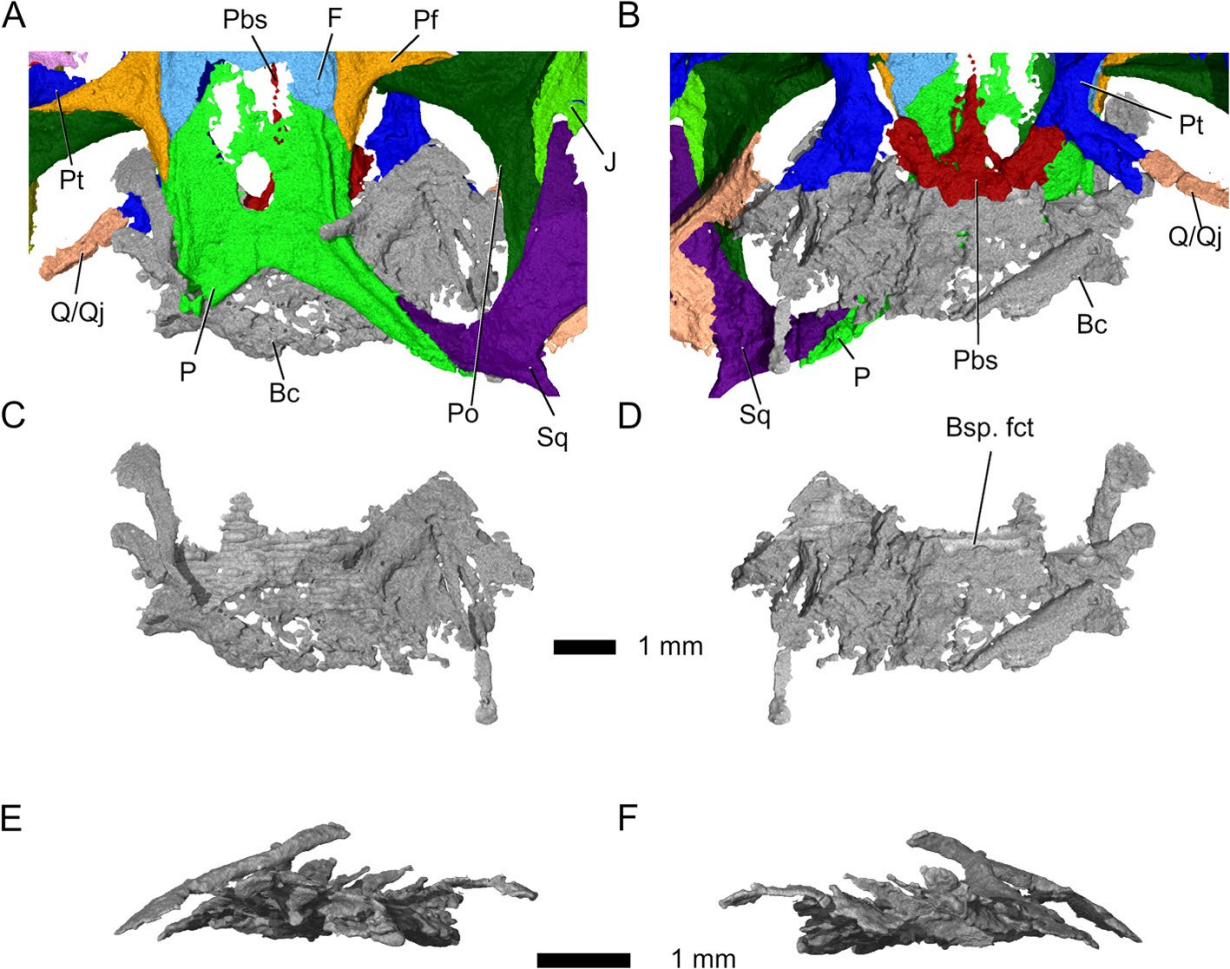
Braincase, **A**-**B** in context, and **C**-**F** isolated. **A** C in dorsal, **B** D ventral, **E** left lateral, and **F** right lateral view. Abbreviations: Bc, braincase; Bsp. fct, basisphenoid facet; F, frontal; P, parietal; Pbs, parabasisphenoid; Pf, prefrontal; Po, postorbital; Pt, pterygoid; Sq, squamosal; Q/Qj, quadrate-quadratojugal complex

**Fig. 19:**
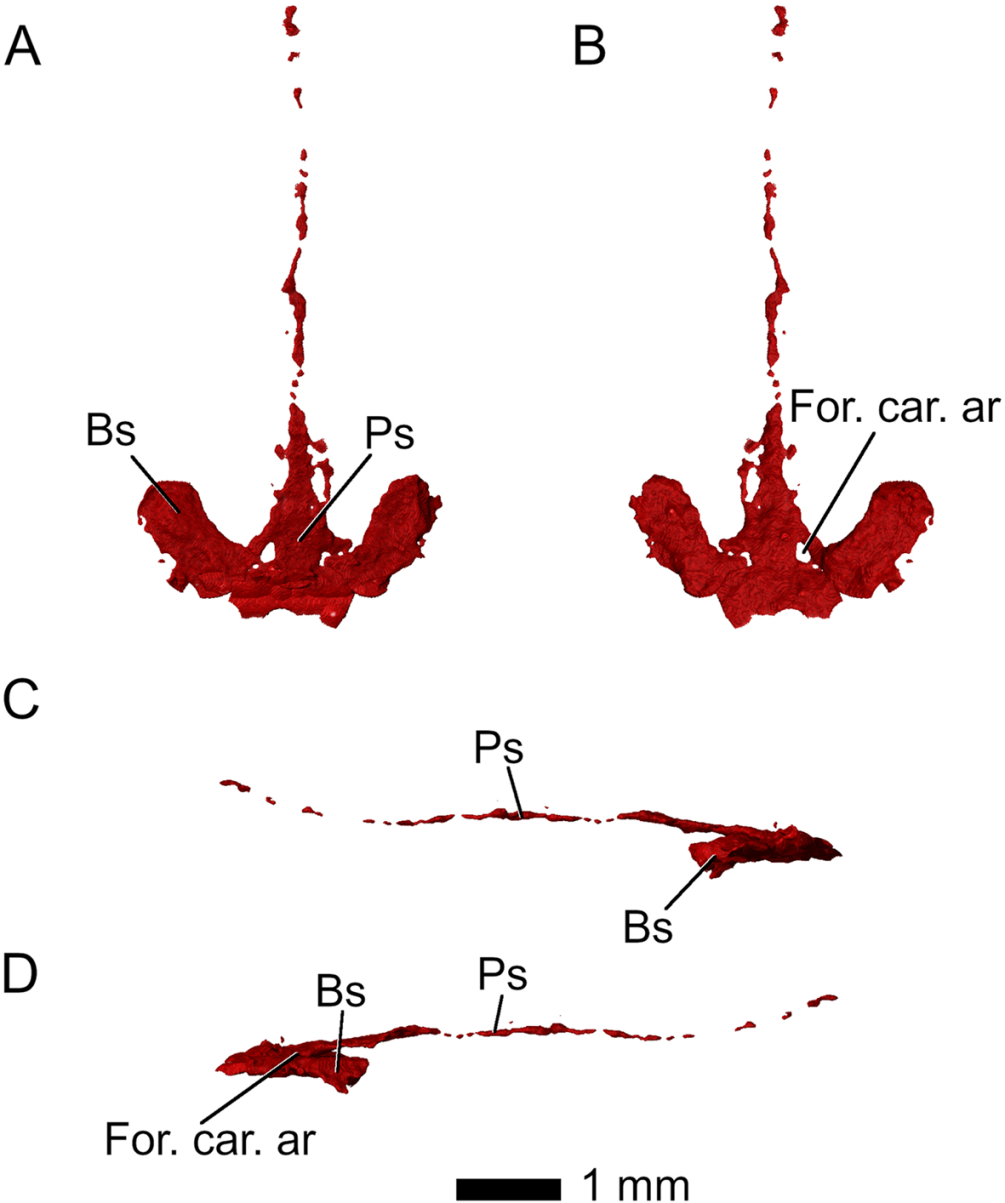
Parabasisphenoid complex in **A** dorsal, **B** ventral, **C** left lateral, and **D** right lateral view. Abbreviations: Bs, basisphenoid; For. car. ar, foramen for internal carotid artery; Ps, parasphenoid

The braincase is preserved but severely distorted, hindering identification of individual elements (Fig. 18). Most of the parasphenoid and basisphenoid is preserved. The bones are almost indistinguishably fused and thus will be discussed as a unit (Fig. 19). A long parasphenoid rostrum extends along the midline of the interpterygoid vacuity from the anterior end of the parabasisphenoid up to the level of the posterior processes of the prefrontals (Fig. 19). The basipterygoid processes articulate with the medial portions of the pterygoid flanges, as in *Diphydontosaurus avonis* [11]. Two openings pierce the parabasisphenoid anteriorly and represent the entrances for the internal carotid artery (Fig. 19A, B).

In *Parvosaurus harudensis*, *epipterygoids* cannot be identified. This could be a consequence of the dorsoventral compression of the specimen.

#### Lower jaw

**Fig. 20:**
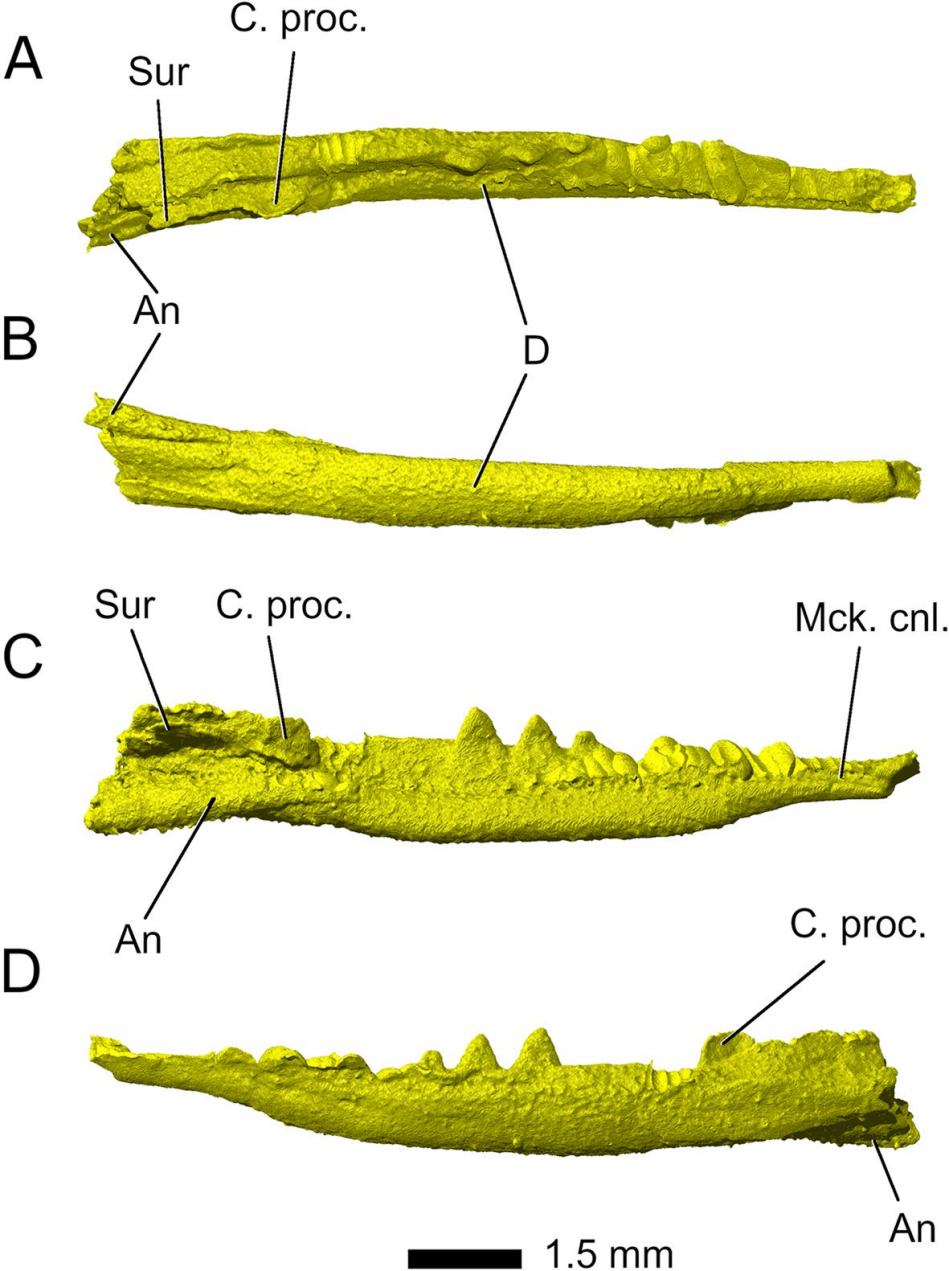
Left mandible in **A** dorsal, **B** ventral, **C** medial, and **D** lateral view. Abbreviations: An, angular; C. proc, coronoid process; D, dentary; Mck. cnl, Meckelian canal; Sur, surangular

The *dentaries* make up the majority of the lower jaws. The body of the dentary is largely complete on the left side but on the right side, only three individual teeth are present with the dentary itself missing. Most of the anterior tooth-bearing section of the left dentary was crushed against the maxilla and palatal bones, so only the three posteriormost teeth and the significant edentulous gap between the posteriormost tooth and the articular complex are visible (Fig. 1). The tooth-bearing portion takes up approximately two thirds of the total length of the preserved lower jaw, with the dentary forming the anterior 90% and the articular complex forming the posterior 10%. The dentary in *Parvosaurus harudensis* is robust and mediolaterally flattened posteriorly. Its preservation makes it difficult to identify the Meckelian canal on the mid-portion of the medial surface of the bone, but it is partially open in the anterior and posterior parts of the dentary (Fig. 20). Due to its state of preservation, no foramina or facets can be identified. No splenial could be identified and the dentary shows no articular facets for it. This indicates the splenial was likely absent, as is typical for rhynchocephalians [18]. There are no clear sutures delimiting the articular, prearticular, surangular, angular, and coronoid*.* The articular complex is separated from the tooth-bearing part of the dentary by an edentulous gap as in *Diphydontosaurus avonis* [11]. The coronoid overlaps the dentary dorsally and forms a prominent coronoid process, which greatly contributes to the medial face of the articular group and is equal in height to that of the body of the dentary at the same position. It extends anteroposteriorly and contacts the surangular ventrally.

#### Dentition

As preserved, the left maxilla bears 18 teeth and the right maxilla, as well as both dentaries, preserve three teeth each. In *Parvosaurus harudensis*, the posterior maxillary teeth have conical crowns with broad bases and pointed apices and sit centred on the jawbone, whereas the anterior maxillary teeth are shorter and slimmer. There is no indication of any ribbing on the teeth. The posteriormost two maxillary teeth are significantly smaller than the more anterior ones, reaching only one third of their height (Fig. 3). The subjacent bigger teeth are very large and conical, much like in *Diphydontosaurus avonis* [11], and decrease in size anteriorly. The teeth in the middle of the maxilla are half as tall as the large posterior teeth but share the overall conical shape. In the anterior third of the maxillary tooth row, the teeth are pin-like, short, and spaced more closely than the more posterior teeth. The left dentary bears only the posterior three teeth, whereas the position of the teeth on the right dentary cannot be determined due to disarticulation with the dentary bone. Interpreting from their shape and size, they likely sit in similar positions on the jawbone as on the left dentary. On both sides, all preserved dentary tooth crowns are conical with a wide base, similar to those in the posterior third of the maxillary tooth row. Posteriorly, the teeth rapidly increase in size, with the posteriormost tooth being twice the height as the anteriormost one. Tooth implantation is classically used as a taxonomically informative tool to identify potential phylogenetic affiliations [36], even though recent findings are painting a much more complex scenario [37]. In the case of sphenodontians, special attention has been given to acrodont dentition (when the teeth are ankylosed to the apex of the jawbone) because outside Sphenodontia, this type of tooth implantation is only found in some basal reptiles such as the captorhinid *Opisthodontosaurus carrolli* [38] and in some squamates. Early rhynchocephalians, however, show other tooth implantation types, with *Gephyrosaurus bridensis* [15] showing only pleurodont and *Diphydontosaurus avonis* showing both pleurodont and acrodont teeth [11]. The transitional aspect of dentition in *Diphydontosaurus avonis* is potentially also found in *Parvosaurus harudensis*. Overall, the dentition pattern in *Parvosaurus harudensis* is almost identical to the pleuro-acrodont dentition in *Diphydontosaurus avonis* [11, 39], which is typical for basal sphenodontians [36]. However, the tooth implantation of *Parvosaurus harudensis* could not be identified due to inadequate resolution of the µCT scans (Fig. S2).

Despite its generally well-preserved dermal skull roof, the holotype of *Parvosaurus harudensis* shows poor ossification in the medial region of the nasal, the anterior region of the parietal including the fronto-parietal suture, and parts of the palate including the palatine (Fig. 1A). In combination with the small size of the skull and the proportionately very large orbits, these features indicate a subadult ontogenetic stage. *Parvosaurus harudensis* also shows additional juvenile characteristics like those proposed for some specimens of *Diphydontosaurus avonis* [11] and *Planocephalosaurus robinsonae* [29], including a broad, flat skull roof and a probably incomplete lower temporal arcade [34]. However, distinction between juvenile and paedomorphic phenotypes is difficult in extinct taxa, and paedomorphosis has been hypothesized for basal rhynchocephalians [18]. Moreover, it is unclear whether the flat skull roof in *Parvosaurus harudensis* has any ontogenetic implications since the specimen was dorsoventrally compressed during fossilization. While showing some juvenile characteristics, the holotype of *Parvosaurus harudensis* also has a fully formed pineal foramen and fully fused parietals as in *Diphydontosaurus avonis* [11] and *Planocephalosaurus robinsonae*, [29] possibly suggesting a later ontogenetic stage. Although the ontogeny of the skull and dentition of rhynchocephalians has been studied in *Sphenodon punctatus* since the early 1900s [40], there is less available research on extinct rhynchocephalians. Published data on extinct taxa (for a review, see [40]) comprise a description of a juvenile *Diphydontosaurus* sp. [39], notes on juvenile features of *Theretairus antiquus*, *Leptosaurus neptunius* [41], *Cynosphenodon huizachalensis* [40] the holotypes of *Pamizinsaurus tlayuaensis* [42] and *Lanceirosphenodon ferigoloi* [43] as well as notes on the dentitions of *Pelecymala robustus*, *Sigmala sigmala*, *Clevosaurus* spp*.*, and *Planocephalosaurus robinsonae* [8, 32, 40]. *Colobops noviportensis*, considered a rhynchosaur by [44], was recently reinterpreted as a juvenile rhynchocephalian [45] as originally proposed by [46]. In the Jurassic *Cynosphenodon huizachalensis*, resembling the condition in *Sphenodon punctatus*, the dentary dentition of hatchlings shows uniform, small anterior teeth and larger posterior teeth of alternating size [40, 47]. This condition is similar to that of the maxillary dentition in *Parvosaurus harudensis*, but information about anterior dentary dentition is lacking. Although there is variation in the sequence, timing and degree of ossification among rhynchocephalians [43, 46–51], there are some consistent points such as the late ossification of the nasal and parietal [52]. It is not possible to identify the exact ontogenetic age of the holotype of *Parvosaurus harudensis*, but we suggest a later ontogenetic stage.

#### Phylogenetic affinity

*Parvosaurus harudensis* was added to the character-taxon matrix of [23] to assess its relationships among rhynchocephalians. The results of both the parsimony and Bayesian analyses were congruent and, in turn, they were also broadly consistent with the results from [23]. *Parvosaurus harudensis* is retrieved as a basal sphenodontian closely related to *Planocephalosaurus robinsonae* and Eusphenodontia (Fig. 2A, S3-S6). Like in [19], *Gephyrosaurus bridensis* was recovered as a basal rhynchocephalian in our analysis, although it was found outside Rhynchocephalia in some topologies published by [23]. Despite their overall resemblance, *Parvosaurus harudensis* and *Diphydontosaurus avonis* do not cluster together as sister taxa in any tree topology recovered in this study (Supplementary Fig. S3-S6).

**Fig. 21:**
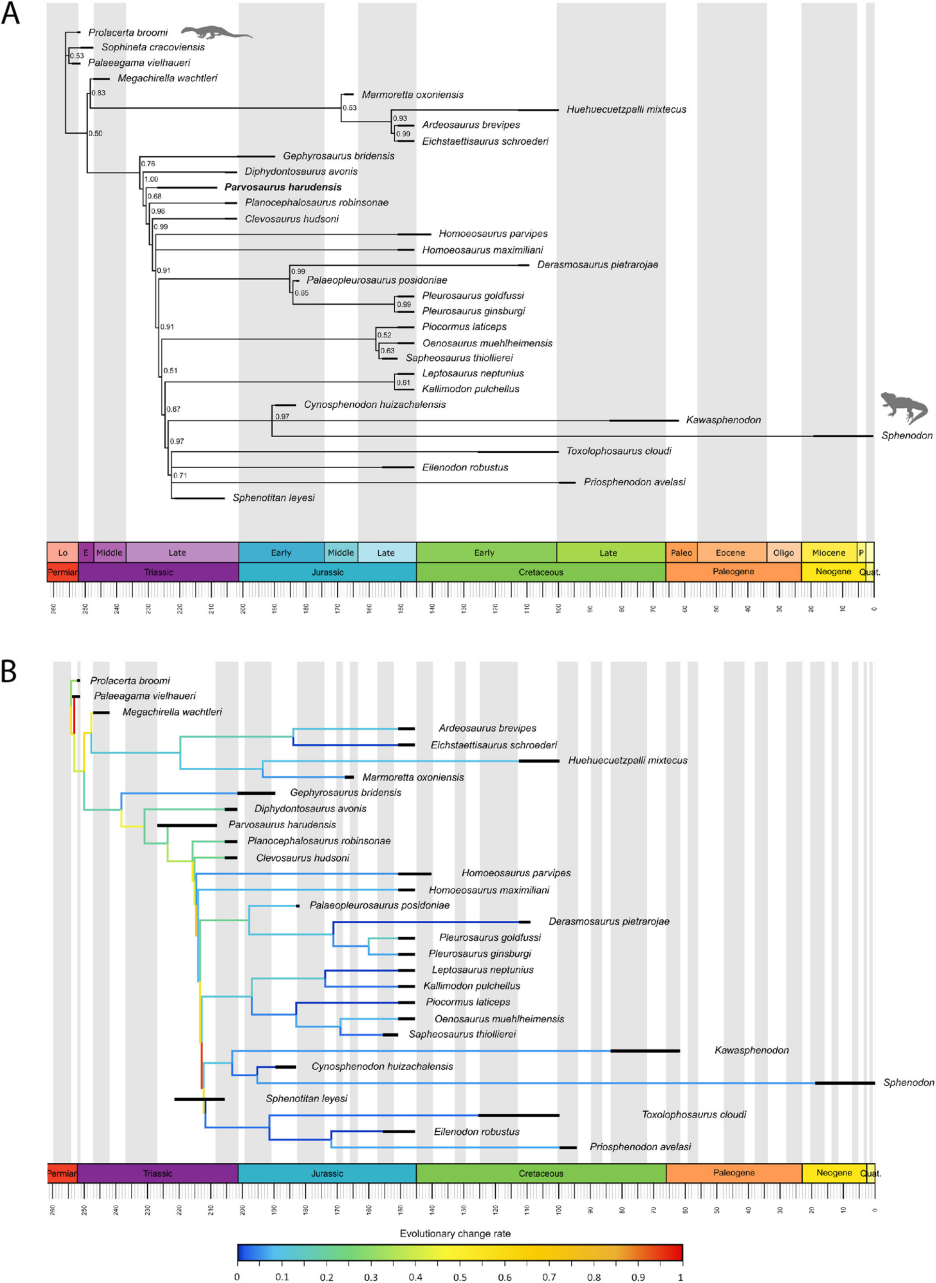
Phylogenetic relationships and evolutionary change rates in Rhynchocephalia. **A** majority-rule consensus phylogeny after the morphological matrix from [23]. Phylogeny is time-calibrated with tip-dates and node ages are calculated under the ‘mbl’ model. Posterior probabilities are given for each node. Gray and white background stripes indicate epoch intervals within each geochronologic period. **B** Evolutionary change rates across Rhynchocephalia based on log-transformed character state changes per million years. Phylogeny is time-calibrated with tip-dates and node ages are calculated under the ‘equal’ calibration model. Gray and white background stripes indicate age intervals within each geochronologic period

In order to understand the impact of the inclusion of *Parvosaurus harudensis* on evolutionary trends among sphenodontians proposed by previous studies [5, 21, 23, 24, 53], an analysis of evolutionary rates was undertaken. For this, two time-calibration approaches (a minimum branch length ‘mbl’ method with the minimum branch length set to 1 myr (see Fig. 21A) and an equal branch length ‘equal’ method with the root length fixed to 2 myr (see Fig. 21B)) were used. These calibration methods were chosen over a Bayesian fossilized birth–death (FBD) model in order to ensure compatibility with the Maximum Likelihood evolutionary rates analysis, which does not allow pre-calibrated trees. Time-calibration of the tree with the ‘mbl’ and ‘equal’ dating methods led to different divergence time estimates for Sphenodontia, Sphenodontinae, and Eilenodontinae, contrasting with the ages calculated by [23]. For instance, for the ‘mbl’ approach, Sphenodontia and sapheosaurs are recovered as being stratigraphically younger, while the divergence times for sphenodontines, eilenodontines, and pleurosaurs (all in the Upper Triassic) are comparable to the dates estimated in their analysis (Fig. 2A). In our study, Sphenodontia and sapheosaurs originate in the Upper Triassic (Carnian and Norian, respectively) in contrast to Sphenodontia diverging in the Permian and sapheosaurs in the Early Jurassic in [23]. The ‘equal’ approach recovers a much earlier divergence time for Sphenodontia (Middle Triassic: Ladinian) and the clade containing Sapheosauridae and *Kallimodon pulchellus* (Upper Triassic: Norian) is much older than suggested by [23] (Fig. 2B). Here, the branch leading to sapheosaurs is much shorter than that found with the ‘mbl’ method. However, eilenodontines are recovered as diverging in the Norian (Upper Triassic) in both the ‘equal’ and ‘mbl’ approaches as well as in the best fit clock model by [23] (see Fig. 21, S7-S9 and [23] Fig. 6a). Although pruning taxa and the choice of clock model may cause some divergences within the phylogeny, they still lie within the previously proposed ranges of divergence estimates [23]. Therefore, we did not include any measures to correct for this divergence.

#### Rates of evolutionary change in sphenodontians

##### Branch rates

Throughout sphenodontian evolution, rates of evolutionary change slow down continuously (Fig. 21B, S7-S11). As found in previous studies [23, 24], basal Sphenodontia had high evolutionary rates, whereas derived taxa display lower overall rates. This general pattern is also recovered in our analysis. Although both studies yield more or less consistently decreasing rates for their datasets, our combination of the phylogeny from [23] with the change rates approach from [24] yields somewhat different results. Both the ‘equal’ and ‘mbl’ approach support previous findings of generally high rates in basal sphenodontians with a trend of decreasing rates toward the crown (Fig. 21B, S7-S9). Within Sphenodontia, the ‘equal’ scaling method yields mid-range rates in *Diphydontosaurus avonis*, *Planocephalosaurus robinsonae,* and *Clevosaurus hudsoni*, but generally lower rates in *Homoeosaurus* spp*.*, pleurosaurs, eilenodontines, and sphenodontines. *Kallimodon* spp., and *Oenosaurus muehlheimensis* retained uncharacteristically high rates, which stands in contrast to the lower rates recovered by [23] and [24]. However, a slight increase in evolutionary rate in *Pleurosaurus goldfussi* can be found in our analysis as well as [23]. Despite differences in the time-scaling, the ‘equal’ and ‘mbl’ approaches recover very similar rates without exception when excluding *Parvosaurus harudensis* from the analysis. In the log-transformed branch rates (see Supplementary Data), the biggest difference between them lies in the internal branch leading from the root to *Prolacerta broomi* with ∆_rate_ = 0.0373. Therefore, the choice of the time scaling approach does not seem to have a profound effect on character state change rates in this dataset. These results suggest that while evolutionary rates drop throughout Sphenodontia as found in previous studies, Sphenodontia experienced a slight trend towards increased rates in some clades during the Jurassic (see *Pleurosaurus goldfussi*, *Kallimodon* spp., and sapheosaurs).

When including *Parvosaurus harudensis* in the analysis, the ‘mbl’ approach recovers basically unchanged rates, with only the branch leading to *Homoeosaurus* spp. yielding much lower rates (Supplementary Fig. S9). However, terminal branches are largely unaffected and therefore illustrate the same trend of decreasing rates throughout sphenodontians. Rates recovered by the ‘equal’ approach, however, are strongly affected by the inclusion of *Parvosaurus harudensis* (Fig. 21B). Since *Parvosaurus harudensis* is the oldest sphenodontian included in the dataset, the ‘equal’ method evens out the spacing between backbone branches by extending branch lengths up to *Parvosaurus harudensis* and compressing the tree on all branches crownward of *Planocephalosaurus robinsonae*. This leads to minimal internal branch lengths and therefore higher rates within Sphenodontidae. Accordingly, terminal branches are recovered with relatively lower rates among sphenodontians. However, even here, a trend of decreasing rates from basal to crownward sphenodontians is recovered. This result yielded with the ‘equal’ calibration approach is more like the results recovered by [24], in which internal branch rates are also significantly elevated compared with terminal branch rates. However, it differs from the result by [23], in which rates are more evenly distributed throughout the phylogeny. Our evolutionary rates analyses thus confirm the previously observed trend of decreasing evolutionary rates within Sphenodontia, but also show increased rates in some taxa, specifically during the Jurassic Period. The addition of *Parvosaurus harudensis* further supports the trend of faster diversification in early but not later sphenodontian evolution.

##### Time rates

**Fig. 22:**
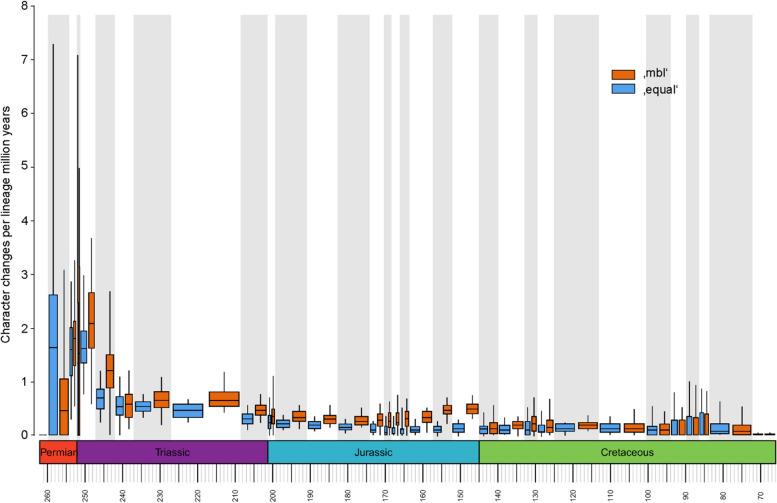
Sphenodontian evolutionary change rates averaged over geochronologic age, including *Parvosaurus harudensis*. Orange, time-calibration using the ‘mbl’ model; blue, time- calibration using the ‘equal’ model. Although Permian and early Triassic rates generally show lower accuracy, Middle and Late Triassic sphenodontians yield overall higher rates than younger Jurassic and Cretaceous sphenodontians in both models. Gray and white background stripes indicate geochronologic ages

The trend toward generally decreasing evolutionary rates from early branching to more highly nested sphenodontians is supported by a rates analysis based on geochronologic age throughout the Mesozoic (Fig. 22, S10). Results show moderate rates in the earliest Triassic with a slight increase during the Carnian and a subsequent decrease in evolutionary rates up to the Late Cretaceous. Instances of elevated rates are found in the Carnian, in the late Early Jurassic, and in the middle Late Cretaceous. The general decrease in rates with isolated, smaller peaks are consistent with patterns found on our topology-based results. Moreover, the high peak at the beginning of the Mesozoic is consistent with an adaptive radiation hypothesis for lepidosauromorphs, and for rhynchocephalians in particular. We also tested for divergences in rates between cranial and postcranial characters and, although cranial characters were more completely scored, we found no significant rate divergences between cranial and postcranial characters (see Supplementary Material).

## Discussion

Although incompletely known, *Parvosaurus harudensis* possesses many features that clearly place it with sphenodontians. Synapomorphies of Sphenodontia (= Sphenodontida [18]) as defined by [11] (i – iii) and by [18] (iv – viii) that are present in *Parvosaurus harudensis* include: (i) acrodont teeth alternating in size in at least part of the dentary and (ii) the maxilla, (iii) posterior process of the dentary extending under the glenoid fossa of the articular complex, (iv) prominent coronoid eminence, (v) palatine further enlarged laterally compared to *Gephyrosaurus bridensis* [15] and suborbital fenestra consequently reduced, (vi) loss of lacrimal, (vii) jugal deeply overlapping quadratojugal laterally and extending well posterior to the mid-length of the lower temporal fenestra, and (viii) jugal contacting squamosal at posterodorsal margin of lower temporal fenestra. Some other common features diagnostic for Sphenodontia on the premaxilla, the palate, and the dentition, including posterolabial or anterolabial flanges on the teeth, could not be confirmed in the holotype of *Parvosaurus harudensis* due to its incompleteness and poor resolution of the µCT data in this area. However, the posterior maxillary teeth are generally conical, closely-spaced, and no border between tooth and the jawbone is visible, while the anteriormost preserved teeth are narrower and pin-like. This indicates that the anteriormost preserved teeth of *Parvosaurus harudensis* might have been pleurodont whereas the posterior teeth might have been acrodont, similar to *Diphydontosaurus avonis*, suggesting it might have been a common feature of basal sphenodontians [36]. This contrasts both with the pleurodont dentition of basal rhynchocephalians including *Gephyrosaurus bridensis* [15] and the exclusively acrodont dentition of more derived sphenodontians [36]. The similar morphologies of these basal taxa, as well as their similar ages and geographical distribution, indicate a generalised sphenodontian skull bauplan was relatively widespread in western Europe during at least the Late Triassic.

The addition of *Parvosaurus harudensis* to the dataset does not significantly affect our understanding of the appearance of major rhynchocephalian traits, as its anatomy corresponds quite closely to the well-established cranial bauplan of the group (Fig. S12). Important rhynchocephalian features such as the absence of the splenial are established before the appearance of *Parvosaurus harudensis*. Likewise, traits such as the fusion of the frontals only arise later, in the origin of Eusphenodontia. Unfortunately, our specimen cannot inform on several other important aspects of rhynchocephalians such as the presence of the mentonian process or the shape of the symphysis.

While *Parvosaurus harudensis* closely resembles *Planocephalosaurus robinsonae* and *Diphydontosaurus avonis*, key anatomical features distinguish it from both species and justify establishment of a new taxon. The maxilla is proportionately more elongate than in *Diphydontosauru*s *avonis* and the posterior teeth are proportionately smaller. The supratemporal fenestra is very large and elongated, and most of its margin is formed by the parietal and postorbital (Fig. S1). Although *Diphydontosaurus avonis* also has a large supratemporal fenestra, the margin of this opening is formed almost in equal parts by the parietal, postfrontal, postorbital, and squamosal. On the other hand, the skull of *Planocephalosaurus robinsonae* is very similar in shape to that of *Parvosaurus harudensis*, but it has a substantially smaller supratemporal fenestra with smaller segments of its margin formed by the parietal and squamosal, respectively. The postorbital of *Parvosaurus harudensis* is ‘Y’-shaped, with a long posterior process and a dorsal process that strongly underlaps the postfrontal, resulting in the oval outline of the supratemporal fenestra. In *Diphydontosaurus avonis,* all three processes of the postorbital are similar in length and the underlapping of the postfrontal is less distinct, whereas *Planocephalosaurus robinsonae* has an almost triangular postorbital that extensively overlaps the postfrontal. In addition, the squamosal in *Parvosaurus harudensis* is wider transversely than that of *Diphydontosaurus avonis* but less so than in *Planocephalosaurus robinsonae*. The quadratojugal, together with the jugal, likely formed an incomplete lower temporal arcade, which is comparable to that in *Diphydontosaurus avonis*. The pineal foramen is oval and more anteriorly placed in *Parvosaurus harudensis*, and the posterolateral processes of the parietal are long and form an acute angle between them. This is more like the condition in *Diphydontosaurus avonis* than in *Planocephalosaurus robinsonae*.

As the differences listed above mostly pertain to relative proportions, it could be argued that the immature ontogenetic stage of the holotype of *Parvosaurus harudensis* can account for these differences. However, the known specimens of both *Diphydontosaurus avonis* and *Planocephalosaurus robinsonae* are likewise not fully grown. Furthermore, we interpret the holotype of *Parvosaurus harudensis* as representing the same, or a slightly more mature, subadult ontogenetic stage than the remains of these two taxa. Maturation in *Sphenodon punctatus* is accompanied by the growth of additional teeth and reduction of the edentulous sections in the maxilla and dentary [47]. Ontogenetic growth is reflected in the marginal tooth-bearing bones of *Sphenodon punctatus* in different ways in the upper and the lower jaw [47]. In the maxilla, growth occurs by the appearance of new, successional teeth both posterior and anterior to the hatchling teeth, which occupies a mid-anterior position once the addition of successional teeth has ceased. However, the maxillary ramus continues to grow anteriorly, eventually creating a small edentulous area between maxillary and premaxillary teeth. The dentary, on the other hand, essentially grows posteriorly, with addition of successional teeth posterior to the hatchling series, which occupies a more anterior portion of the dentary once successional teeth stop appearing. The dentary also continues to grow posteriorly to create an edentulous region between the last successional tooth and the coronoid process. The presence of an edentulous region in the dentary is also present in agamid squamates [47, 54], which suggests that this may have been a plesiomorphic feature of lepidosaurs. The growth pattern in the maxilla, however, may be exclusive to sphenodontians. The edentulous region is definitely present on the dentary of *Parvosaurus harudensis* and, even though the maxilla is damaged anteriorly, a short portion of the anteriormost region of the maxillary ramus also seems to lack teeth. In *Diphydontosaurus avonis* [11] the dentary also shows an edentulous region between the tooth row and the coronoid eminence. However, the maxillary tooth row extends to the anteriormost region of the maxilla. The same is also true for *Planocephalosaurus robinsonae* [29], with the difference that the maxilla shows a very small anterior region without teeth. The pattern seen in *Diphydontosaurus avonis* and *Planocephalosaurus robinsonae*, however, does not seem to match entirely with that observed in *Sphenodon punctatus.* In the latter, the addition of successional teeth anterior to the hatchling teeth and the growth of the maxilla further anteriorly takes place before the development of the edentulous region in the dentary. Therefore, the presence of this region in the dentary of *Diphydontosaurus avonis* and *Planocephalosaurus robinsonae,* but the lack of a similar region in the maxilla is puzzling. A detailed review of the growth pattern in the dentition and jaws of sphenodontians is out of the scope of this work, but if we are to accept the maxillary diastema of *Parvosaurus harudensis*, we may consider its known specimen is slightly more mature than both *Diphydontosaurus avonis* and *Planocephalosaurus robinsonae*. If we do not accept this feature of *Parvosaurus harudensis*, then it should be considered in an equivalent ontogenetic stage as the other two taxa. In any case, the proportions listed above in the skull of *Parvosaurus harudensis* are not likely to drastically change in relation to *Diphydontosaurus avonis* and *Planocephalosaurus robinsonae*, still serving as good distinguishing features between all these taxa. Additionally, the final maxillary tooth count of *Parvosaurus harudensis* seems to be slightly lower than *Diphydontosaurus avonis*, which may suggest a gradual decrease in the overall tooth count in sphenodontians.

The holotype of *Parvosaurus harudensis*, with a total skull length of 16 mm, is a particularly small specimen, but comparable in size to other Triassic basal rhynchocephalians, which were generally small in comparison to some geologically younger taxa [55]. Although sphenodontians were generally small in the Mesozoic, poor ossification of the nasals and of the anterior portion of the parietal, as well as the proportionately very large orbits, suggest MB.R.4520.2 was an immature specimen. These ossification patterns in the dermal skull roof are similar to the ones of *Colobops noviportensis* [45], which has been described as a juvenile clevosaurid. However, it is also possible that dwarf phenotypes were present among basal sphenodontians such as *Parvosaurus harudensis*, leading small-sized individuals to retain juvenile-like skull proportions. This would have broad implications for the basal sphenodontian ecology, but further studies are needed to explore this issue in detail.

The discovery of *Parvosaurus harudensis* not only increases the Triassic fossil record of sphenodontians, but also starts filling the ~ 60 myr temporal gap in their European fossil record and therefore helps to fill in the extensive ghost lineage in early Sphenodontia. *Parvosaurus* represents the first nearly complete skull of a basal sphenodontian from the middle to late Norian (227 – 208 myr) of Germany, making it the oldest specimen of this kind in Europe [11], and one of the oldest in the world [34; 45]. In terms of the known sphenodontian record, in continental Europe it is predated only by the Middle Triassic (Ladinian: Longobardian) *Wirtembergia hauboldae* [19, 56] (late Ladinian: Longobardian, 239–240 myr) from the Vellberg locality in Baden-Württemberg (Germany). Worldwide, it is also one of the oldest known sphenodontian occurrences, together with *Clevosaurus brasiliensis*, *Lanceirosphenodon ferigoloi,* and *Microsphenodon bonapartei* from Brazil [35, 43, 57], *Sphenotitan leyesi* from Argentina [10], and *Colobops noviportensis*, *Micromenodon pitti*, *Trullidens purgatoiri*, as well as *Paleollanosaurus fraseri* from the United States [44, 45, 58–60]. While there are some European sphenodontians from the Norian and older (*Diphydontosaurus* sp. from Italy [39], *Brachyrhinodon taylori* from Scotland [61, 62]), most are from the Rhaetian or younger. This indicates that the general skull morphotype observed in early sphenodontians was established around 10 myr earlier than previously thought, since *Parvosaurus harudensis* and *Diphydontosaurus avonis* share a number of anatomical similarities. Conversely, these similarities, together with their somewhat close phylogenetic affiliations could both potentially give additional support for an older date for the Tytherington Quarry [63]. In continental Europe, most sphenodontian records do not appear until the Late Jurassic, when several taxa are recorded from France and especially Germany, such as *Homoeosaurus* spp. and *Sapheosaurus thiollierei* [64]. This makes *Parvosaurus harudensis* especially valuable for research on the origin and early evolution of the sphenodontian skull bauplan, and further supports the hypothesis of an early sphenodontian radiation throughout Europe.

While helping fill in this gap in the early evolutionary history of sphenodontians, our analyses including *Parvosaurus harudensis* also alters divergence times for some sphenodontian clades. For instance, the addition of *Parvosaurus harudensis* to our analysis not only reconsiders the status of *Gephyrosaurus bridensis* as a basal rhynchocephalian, but also produces, depending on the model adopted, an early to middle Middle Triassic estimate for the divergence time between Sphenodontia and Squamata, around 240 Ma. This is in accordance with the fossil record described above and [56], but contrasts [23], who finds an older, Early Triassic age for the origin of this group, at least 10 myr earlier than our results – and possibly earlier, in the Permian. Likewise, our findings show a mid-Late Triassic age for the origin of Eusphenodontia under the “equal” model, around 215 Ma, while [23] find this to take place in the Middle Triassic (240 Ma), or 25 myr before ours. This is more similar to our results using the ‘mbl’ model, which yields a divergence time around 230 Ma for this clade. *Parvosaurus harudensis* belongs to a later-diverging lineage than some other well-known sphenodontians, especially *Diphydontosaurus avonis*, while also being older. This may indicate that older divergence estimates for some early sphenodontian clades may be more accurate; however, that does not refute the hypothesis of true exceptionally high early evolutionary rates characteristic of adaptive radiation events. The clarification of such conflicting possibilities is unfortunately still pending more fossil discoveries.

The long rhynchocephalian and squamate ghost lineage poses the risk of being vulnerable to potential methodological biases towards internal branches, possibly affecting evolutionary rates. While our approach to explore evolutionary change rates through the ‘equal’ and ‘mbl’ dating methods might introduce shorter branches towards the root of the tree, artificially elevating rates in the Early Triassic, more information is available on taxa within Rhynchocephalia in our dataset, stabilising the evolutionary rates within the clade. Divergence-time estimates in our study are mostly in agreement with other studies [23, 53], and therefore leave us confident in the statistical significance and meaningfulness of our results.

Sphenodontians were quite common in the Mesozoic of Europe, and basal taxa have been found on the British Isles (e.g. [11, 29]), Italy [65] and Germany (e.g. [16, 56]). During the Mesozoic, up to their extinction in Laurasia [5, 14] and their second global extinction event at the K-Pg boundary [66, 67], they spread worldwide, with only a few taxa remaining in South America (*Kawasphenodon expectatus* [55]) and New Zealand (*Sphenodon punctatus*) from the Paleogene onwards. However, Triassic sphenodontians are only sparsely represented in the fossil record of Germany (*Polysphenodon muelleri* [9], *Diphydontosaurus* sp. [39], *Wirtembergia hauboldae* [19]). With its subadult ontogenetic stage and phylogenetic position in basal Sphenodontia, *Parvosaurus harudensis* not only suggests a European origin of sphenodontians but also offers new insights into early sphenodontian anatomy and a generalised basal sphenodontian skull bauplan. This led to renewed interest in the evolutionary change rates of early sphenodontians. The inclusion of *Parvosaurus harudensis* in the character-taxon matrix by [23] did not significantly change evolutionary rates found therein but does paint a more nuanced picture of the early evolutionary history of the group. Depending on the time scaling model used, the inclusion of *Parvosaurus harudensis* significantly pushes back divergence times for more derived sphenodontians, and therefore elevates rates in branches leading towards the crown. Although divergence time estimates for sphenodontians, and specifically eusphenodontians, were considerably affected by the inclusion of the new taxon, a general trend of decreasing rates could be still observed over the course of the Mesozoic. Our analyses, however, were able to identify reversals in this trend, indicating additional small peaks of elevated evolutionary change throughout the Jurassic and Cretaceous, which somewhat opposes the pattern retrieved by [23] and [56].

The decrease in evolutionary rates is more dramatic in terminal branches, but the consistently elevated rates of the internal ones also eventually fall in our analyses. [23] recovered a continuous rate drop throughout Sphenodontia, with basal branches like the ones leading to *Diphydontosaurus avonis*, *Planocephalosaurus robinsonae,* and Clevosauridae, and Eusphenodontia having the highest rates. This is similar to the results found here, except the addition of *Parvosaurus harudensis* is responsible for a slight decrease in these rates – which, given the similarity between its anatomy and that of *Diphydontosaurus avonis*, is not surprising. The present study is in agreement with [24] in showing more heterogeneous rates overall, with similar peaks of evolutionary rates. However, the rates remain higher in the internal branches throughout the topology, with only localised decreases in some terminal branches. Our results thus conciliate both previous analyses [23, 24] in that they recognise moderately high but decreasing rates in evolutionary changes throughout the evolutionary history of sphenodontians. However, a more recent study on lepidosaurian evolutionary rates [21], while recovering high rates within Sphenodontia overall, yields steadily increasing evolutionary rates of body size within the clade. Therefore, while slowing down the diversification of morphotypes throughout the Mesozoic, sphenodontians might simultaneously have undergone more changes in body size. While elevated evolutionary rates could have contributed to sphenodontian extinction, further studies on the link between evolutionary rate and clade survival are needed. Adding recently described sphenodontian material [35, 45, 59, 60, 68] to the phylogenetic matrix and conducting an evolutionary rates study including morphotype as well as body size measurements in future analyses will help understand early sphenodontian history even better.

## Conclusions

Despite some recent discoveries, the early evolutionary history of Sphenodontia remains poorly understood, especially when considered from a chronological perspective. Although according to the most recent estimates the origin of the group can be traced back to the Permian–Triassic boundary [30, 53, 69], the European Triassic fossil record of basal sphenodontians is still poor, being mostly represented by fragmentary remains [56]. Except for *Diphydontosaurus avonis* [11], most information about early sphenodontians comes from the Jurassic of England [15, 70, 71]. The discovery of the almost complete skull of *Parvosaurus harudensis* from the Norian of Germany thus represents an important addition to the fossil record of the group. It also represents the oldest articulated sphenodontian skull from Europe and one of the oldest in the world. Anatomically, *Parvosaurus harudensis* closely resembles *Diphydontosaurus avonis*, but can still be distinguished from it by, for instance, a more elongated maxilla, larger supratemporal fenestra, and equal participation of the jugal and squamosal in the upper temporal bar. Whereas the holotype of *Parvosaurus harudensis* is a relatively small specimen, we do not consider it a juvenile but rather a subadult specimen, so that these relative proportions were not likely to change significantly as the animal matured further. Recovery of *Parvosaurus harudensis* as closely related to *Planocephalosaurus robinsonae* and to Eusphenodontia in our phylogenetic analyses reinforces its distinct taxonomic status. It also shows that the general cranial phenotype of early sphenodontians was established about 10 myr earlier than thought. The inclusion of *Parvosaurus harudensis* in an evolutionary rates analysis reconciles previous contrasting attempts to assess morphological changes in the early history of Sphenodontia, which found either high but decreasing rates or high rates with peaks in changes. Here, we recover high rates with peaks for the basal branches, while showing a trend of generally decreasing rates towards more crownward and nested branches. When analysed from a temporal point of view, the Triassic represents the time interval with the highest evolutionary rates for sphenodontians, with other, lower peaks in the Jurassic and Cretaceous. The high peak in the earliest Triassic supports previous models of adaptive radiation of lepidosauromorphs. *Parvosaurus harudensis* thus paints a more complex scenario in the early evolutionary history of sphenodontians.

## Methods

### Computed tomography

The holotype MB.R.4520.2 is housed in the fossil reptile collection of the Museum für Naturkunde in Berlin (Germany). µCT scans were performed in house using a Phoenix|x-ray Nanotom S tomography machine (GE Sensing and Inspection Technologies GmbH, Wunstorf, Germany) with a voltage of 110 kV and a current of 150 µA, capturing 2,000 images with an exposure time of 750 ms and a resolution of 10.23 µm. Slices were reconstructed using the datos|x v.2.3.0.844—RTM reconstruction software (GE Sensing and Inspection Technologies GmbH, Phoenix—x-ray) and the resulting volume was manually segmented and analysed in VG Studio Max 3.3 (Volume Graphics GmbH, Heidelberg, Germany) at the 3D-Visualisation Laboratory at the Museum of Natural History Berlin using the region grower and pen tools.

### Time-calibrated phylogenetic analysis

A sphenodontian phylogeny was constructed to explore the relationship of *Parvosaurus harudensis* within Rhynchocephalia using the morphological matrix by [23]. In the analysis performed by [24], incompletely resolved phylogenies are prepared for the change rates analyses by breaking all polytomies at random, inevitably losing phylogenetic information. We solved this problem by running preliminary analyses and identifying rogue taxa to be excluded a priori, thus resolving all polytomies based on highest likelihood. *Sophineta cracoviensis* and the *Clevosaurus* complex (except for *C. hudsoni*) were pruned from the parsimony phylogenetic and evolutionary rates analyses. This, with the addition of *Parvosaurus harudensis*, resulted in a matrix comprising of 30 taxa and 131 characters.

To provide a timescale for the phylogeny, first and last appearance dates (FAD and LAD, respectively) were compiled for each taxon from the Paleobiology Database (see Supplementary Data). The age of *Parvosaurus harudensis* is based on the Arnstadt Formation in which it occurs, and so the upper and lower boundaries of the Norian were used as LAD and FAD, respectively. Two different dating approaches were performed to facilitate assessment of the stability of the time-scaling and evolutionary rates methods. Following the methods and settings recommended by [72] and [73], branch lengths were computed in R v.4.1.0 (R Core Team, 2021) using the ‘equal’ and ‘minimum branch length’ [mbl] methods. For the ‘equal’ method, the root-length was fixed to a 2 myr duration. For the ‘mbl’ method, it was set to a minimum duration of 1 myr. This way of time-calibration was chosen over a Fossilised Birth Death model through Bayesian Inference to allow for more direct comparison to the evolutionary change rates analysis performed by [24]. In order to track the influence of *Parvosaurus harudensis* on the phylogeny and evolutionary change rates, all analyses were performed twice, once including, and once excluding *Parvosaurus harudensis.*

### Bayesian analysis

The phylogenetic analysis was performed with the settings from [23] under the Mk model [74], applying a gamma distribution for variable rates. No outgroup was set. The analysis was run for 10^7^ generations with 2 runs for 4 chains each. Parameters were sampled for every 1,000 generations and relative burn-in was set to 50%. Posterior node probabilities for all Bayesian phylogenies were exported from FigTree v.1.4.4. All recovered phylogenies can be viewed in the Supplementary Data.

### Maximum parsimony analysis

A phylogenetic maximum parsimony analysis was performed in TNT v.1.5 [75] under implied weights, with a concavity index (K) = 3 (all analyses performed with higher k -values resulted in the same singular tree). As for the Maximum Likelihood rates analyses, a single, fully resolved tree was beneficial, Parsimony was preferred as input for the evolutionary rates analyses over Bayesian Inference methods as seen in [23]. All branches were collapsed to zero length prior to the analysis. Trees were searched using 1,000 replications of Wagner trees with a single random seed and Tree Bisection Reconnection (TBR), saving 10 trees per each of the 1,000 replications. This resulted in the output of a single, fully resolved tree with a fit of 21.80595. Character state changes were counted for each branch using TNT v.1.5 and mapped on the phylogeny using the “Branch length” function (Fig. S11).

### Evolutionary rates analysis

Evolutionary rates were assessed using Maximum Likelihood methods following the protocols of [24, 73, 74, 76, 77]. The time-calibration and evolutionary rates analyses were performed in R v.4.1.0 (R Core Team, 2021) with the Claddis [77], paleotree [78] and tidyverse [79] packages with a modified version of the script by [24] and [73]. All analyses were repeated and averaged over 100 repetitions. To address uncertainty arising from sampling each terminal taxon’s age, node ages were randomised for each of the 100 replicates within the range of estimated ages. LRT significance testing was chosen over AIC for better comparability to the script by [24] with an alpha threshold of 0.01 used to evaluate significance, and with Benjamini–Hochberg false discovery rate correction. Partitions were used for time-bins, character partitions, clade partitions and branch partitions (see Supplementary Data). Time-bins were partitioned into geochronologic ages ranging from the Wuchiapingian (upper Permian) to the Maastrichtian (Late Cretaceous). Characters were partitioned into cranial and postcranial characters. Evolutionary rates of the time-calibrated phylogeny were assessed over 100 runs with randomised dates and the resulting rates were illustrated by averaging over the 100 runs.

### Supplementary Information

**Supplementary Material 1.**

## Data Availability

Supplementary material includes: 1] Bayesian phylogenetic analysis; 2] Maximum Parsimony phylogenetic analysis; 3] R script for the evolutionary rates analysis; 4] input and output data and calculations made for the phylogenetic and rates analyses; 5] a PDF file containing additional figures showing anatomical features, phylogenetic results and evolutionary rates results; 6] the original µCT dataset; 7] the reconstructed 3D surface models. All supplementary materials can be downloaded from: 10.5061/dryad.280gb5mwd.
