# Supplementary material for: A new sphenodontian (Diapsida: Lepidosauria) from the Upper Triassic (Norian) of Germany and its implications for the mode of sphenodontian evolution
Source: BMC Ecol Evol. 2024 Mar 16;24:35. doi: 10.1186/s12862-024-02218-1 (PMC10944618; doi:10.1186/s12862-024-02218-1)
Supplement: Supplementary file 1 — Supplementary Material 1. [file 12862_2024_2218_MOESM1_ESM.docx]

SUPPLEMENTARY FIGURES

Freisem, L. S.; Müller, J.; Sues, H.-D.; Sobral, G. - A new sphenodontian (Diapsida: Lepidosauria) from the Upper Triassic of Germany and its implications for mode of sphenodontian evolution

All correspondence to [lisa.freisem@gmail.com](mailto:lisa.freisem@gmail.com) and [gabriela.sobral@smns-bw.de](mailto:gabriela.sobral@smns-bw.de)


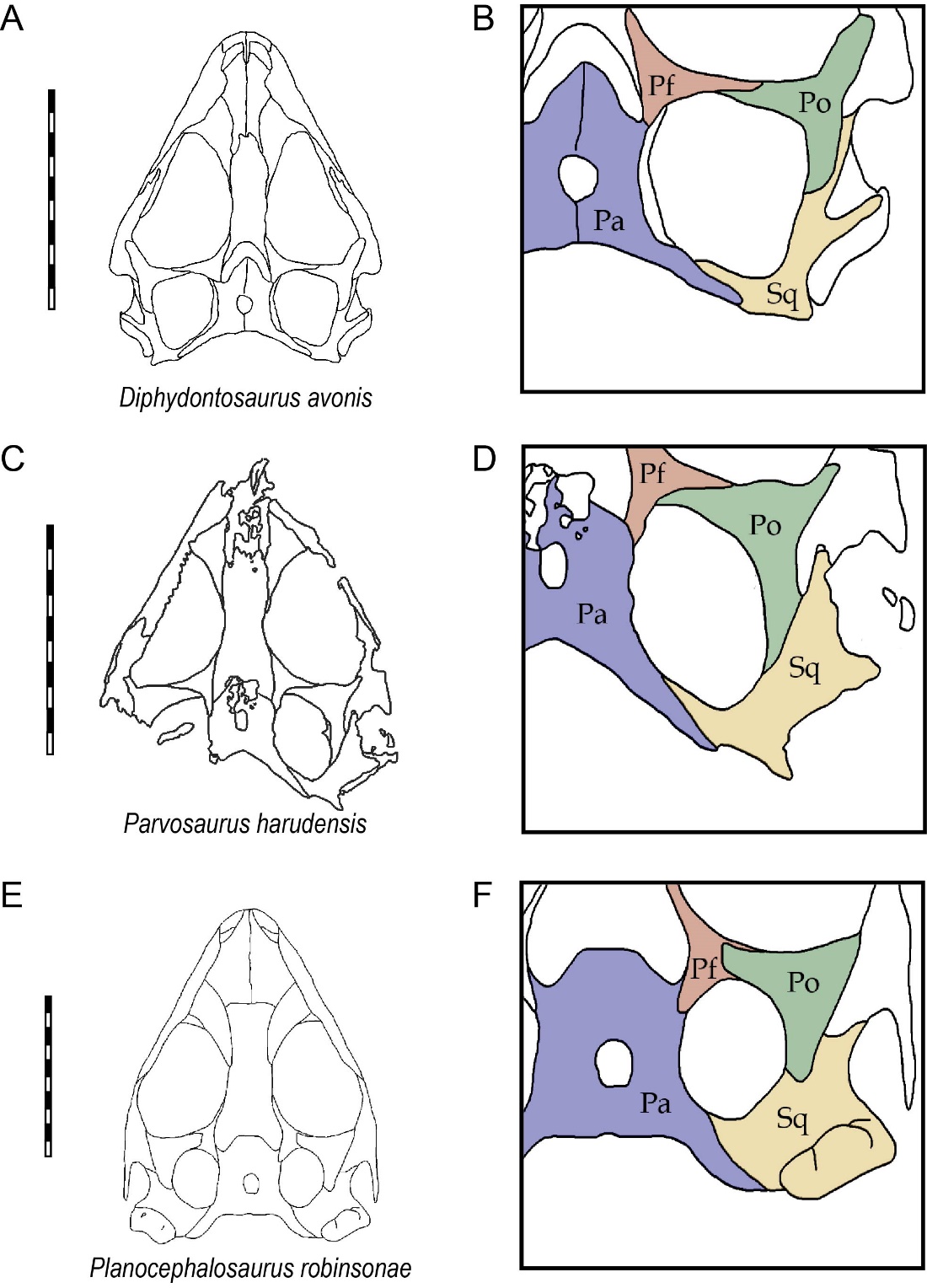


Fig. S1. Comparison of dermal skull roof shape (A, C, E) and shape of the supratemporal fenestra (B, D, F) between *Diphydontosaurus avonis* (after reconstruction by Whiteside, 1986 [11])*,* *Parvosaurus harudensis*, and *Planocephalosaurus robinsonae* (after reconstruction by Fraser, 1982 [30]) in dorsal view. Scale bar equals 1 cm. Abbreviations: Pa, parietal; Pf, postfrontal; Po, postorbital; Sq, squamosal.


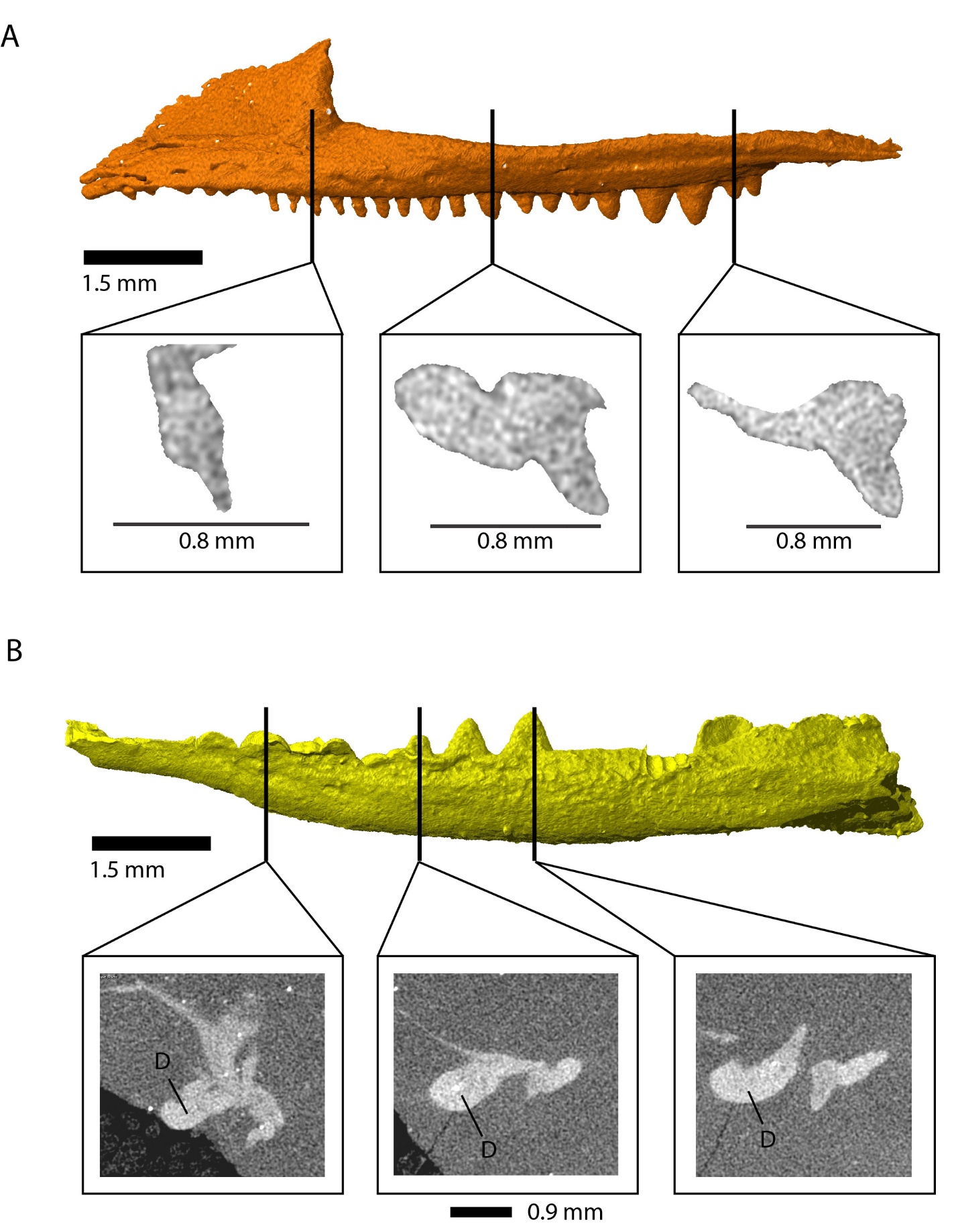


Fig. S2. Cross-sections of A, maxillary and B, dentary dentition in *Parvosaurus harudensis.* Snapshots were taken from the segmented (A) and raw (B) µCT data. Abbreviation: D, dentary.


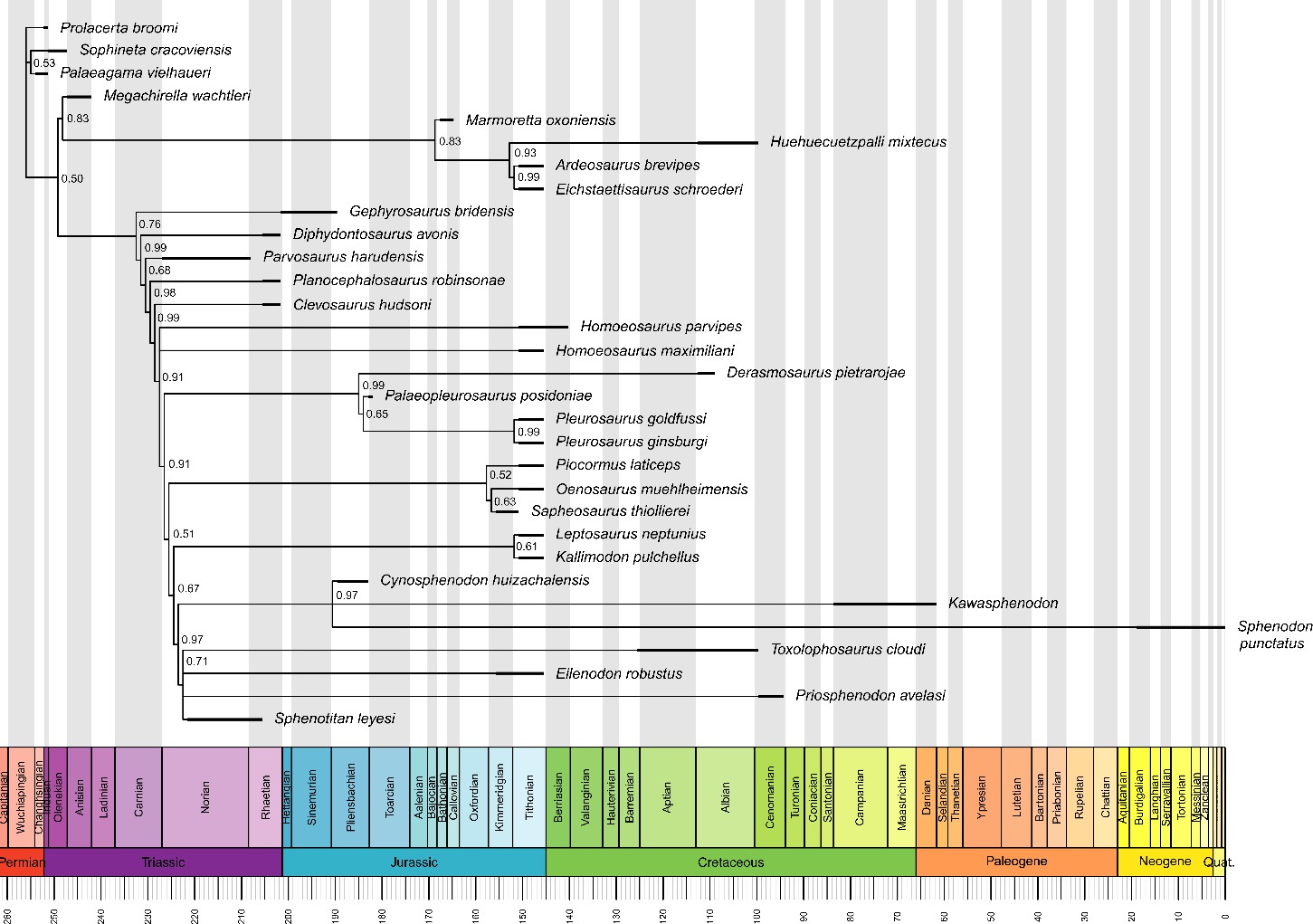


Fig. S3. Majority-rule phylogeny showing possible sphenodontian relationships and the phylogenetic position of *Parvosaurus harudensis*. Time-calibration was performed using tip- dates under the ‘mbl’ model. Grey and white background stripes indicate age intervals within each geochronological period.


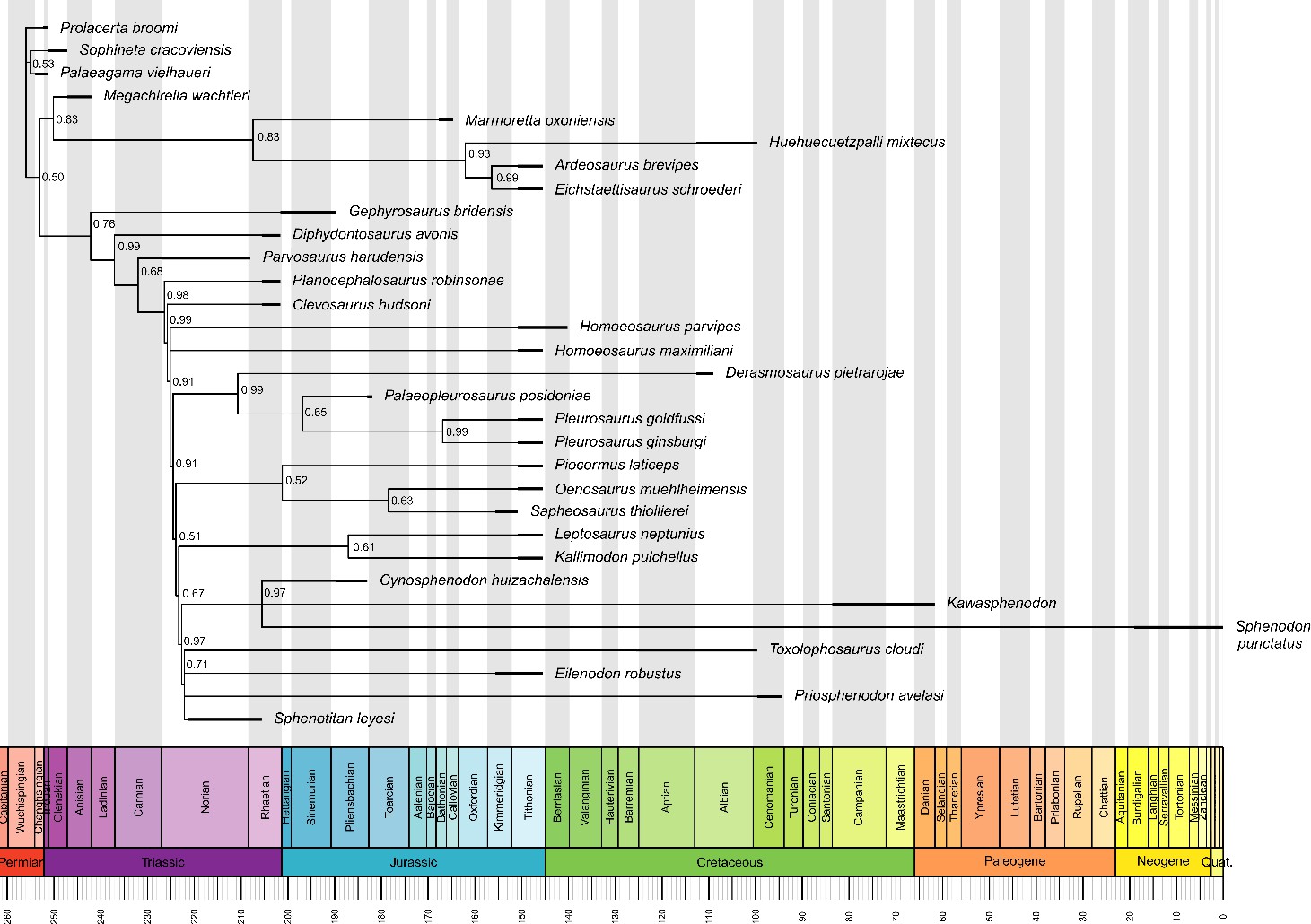


Fig. S4. Majority-rule phylogeny showing possible sphenodontian relationships and the phylogenetic position of *Parvosaurus harudensis*. Time-calibration was performed using tip- dates under the ‘equal’ model. Grey and white background stripes indicate age intervals within each geochronological period.


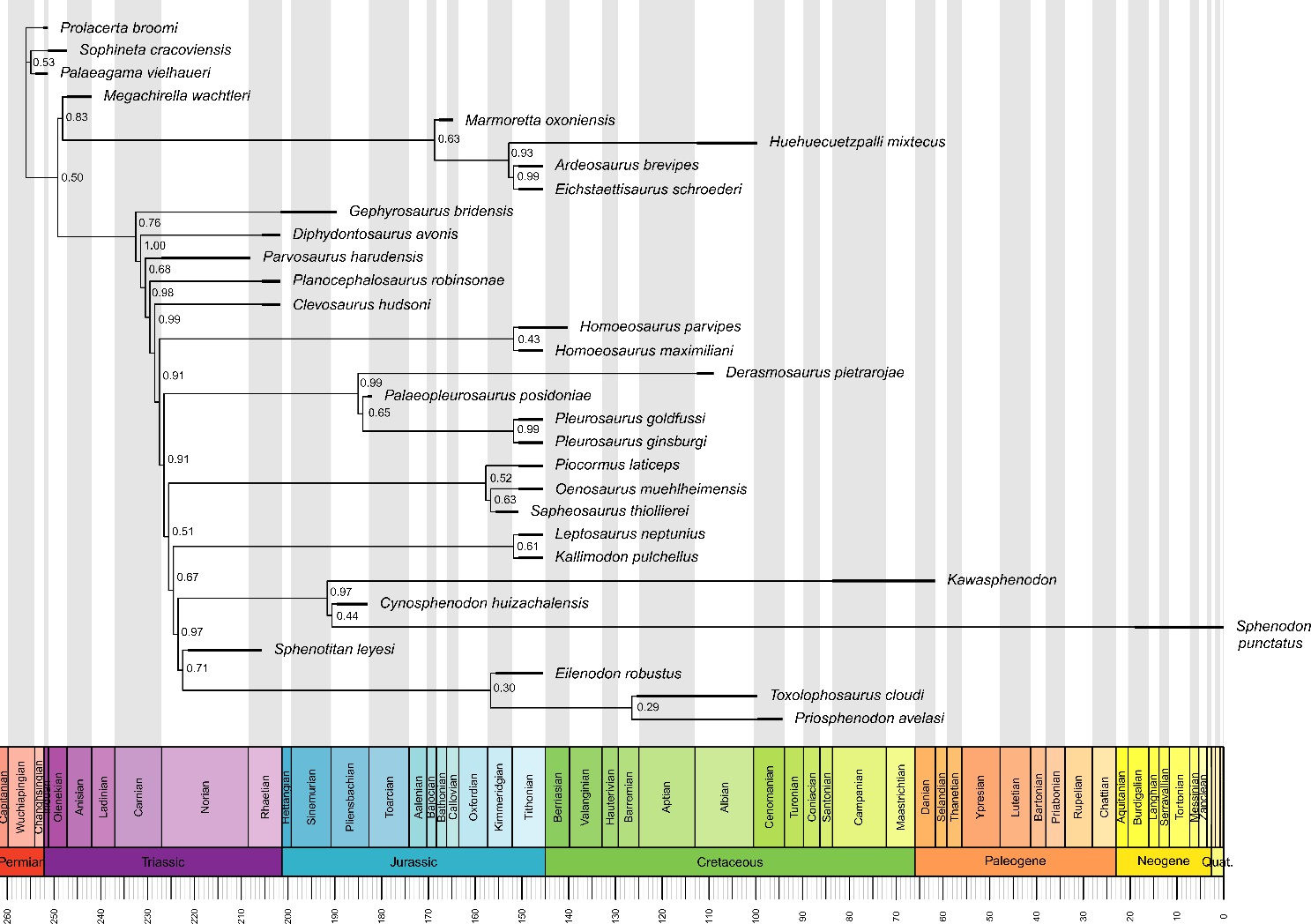


Fig. S5. All-compatibility phylogeny showing possible sphenodontian relationships and the phylogenetic position of *Parvosaurus harudensis*. Time-calibration was performed using tip- dates under the ‘mbl’ model. Grey and white background stripes indicate age intervals within each geochronological period.


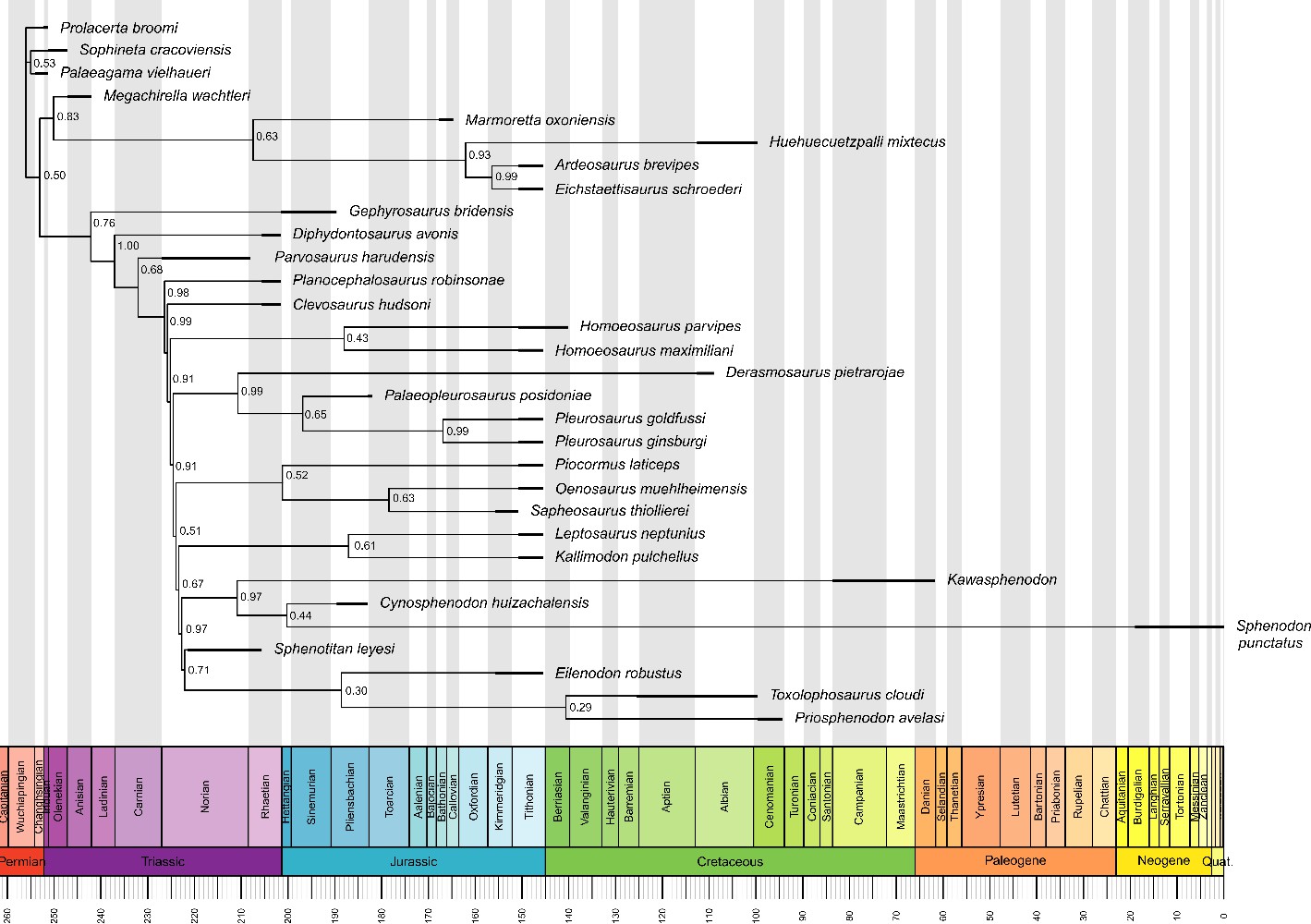


Fig. S6. All-compatibility phylogeny showing possible sphenodontian relationships and the phylogenetic position of *Parvosaurus harudensis*. Time-calibration was performed using tip- dates under the ‘equal’ model. Grey and white background stripes indicate age intervals within each geochronological period.


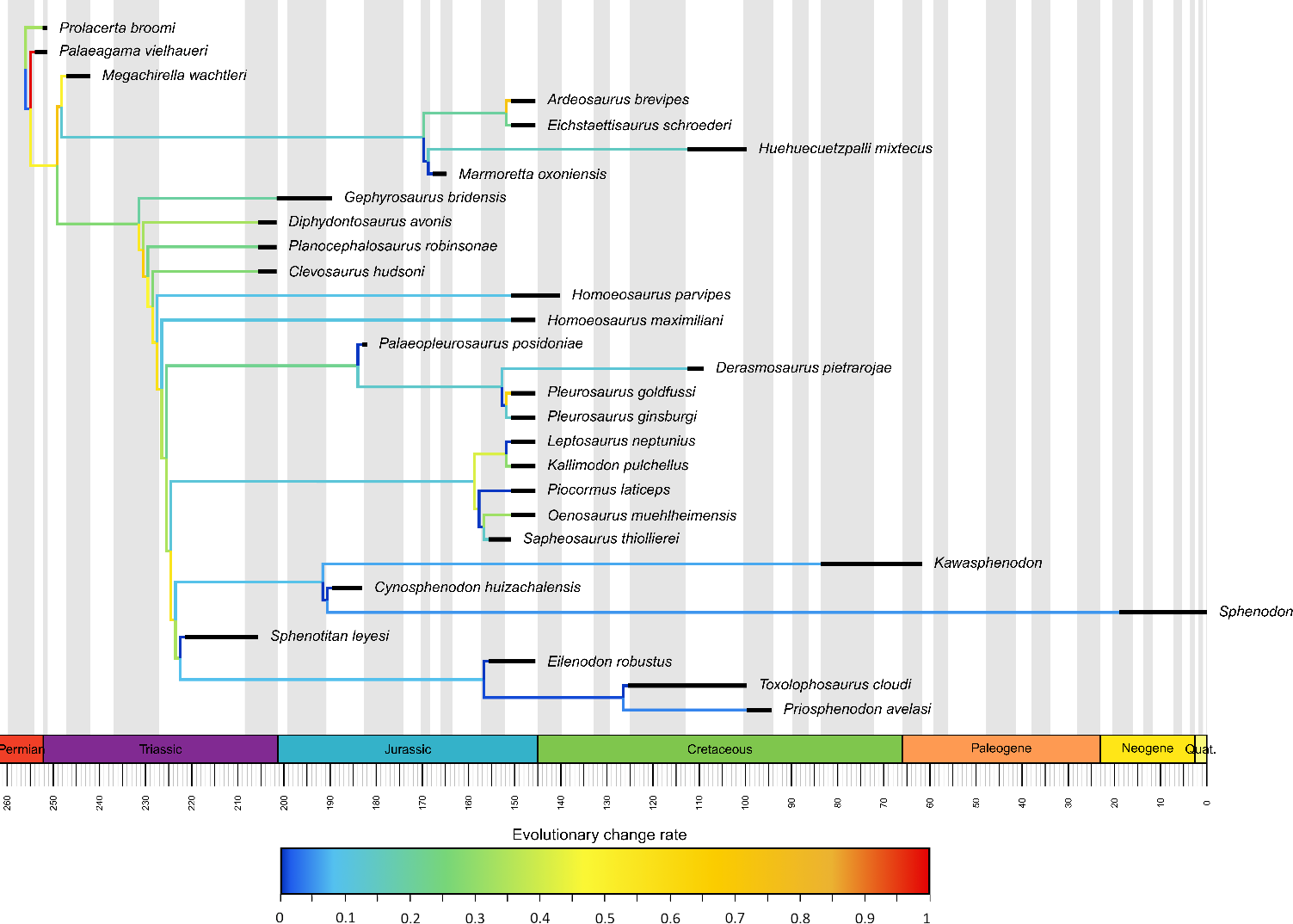


Fig. S7. Evolutionary change rates calculated through character state changes per million years using tip-dates and calculating node ages under the ‘mbl’ time-calibration model, excluding *Parvosaurus harudensis*. Rates are log-transformed for easier visualization. Basal sphenodontians consistently show higher rates than derived taxa. Grey and white background stripes indicate age intervals within each geochronological period.


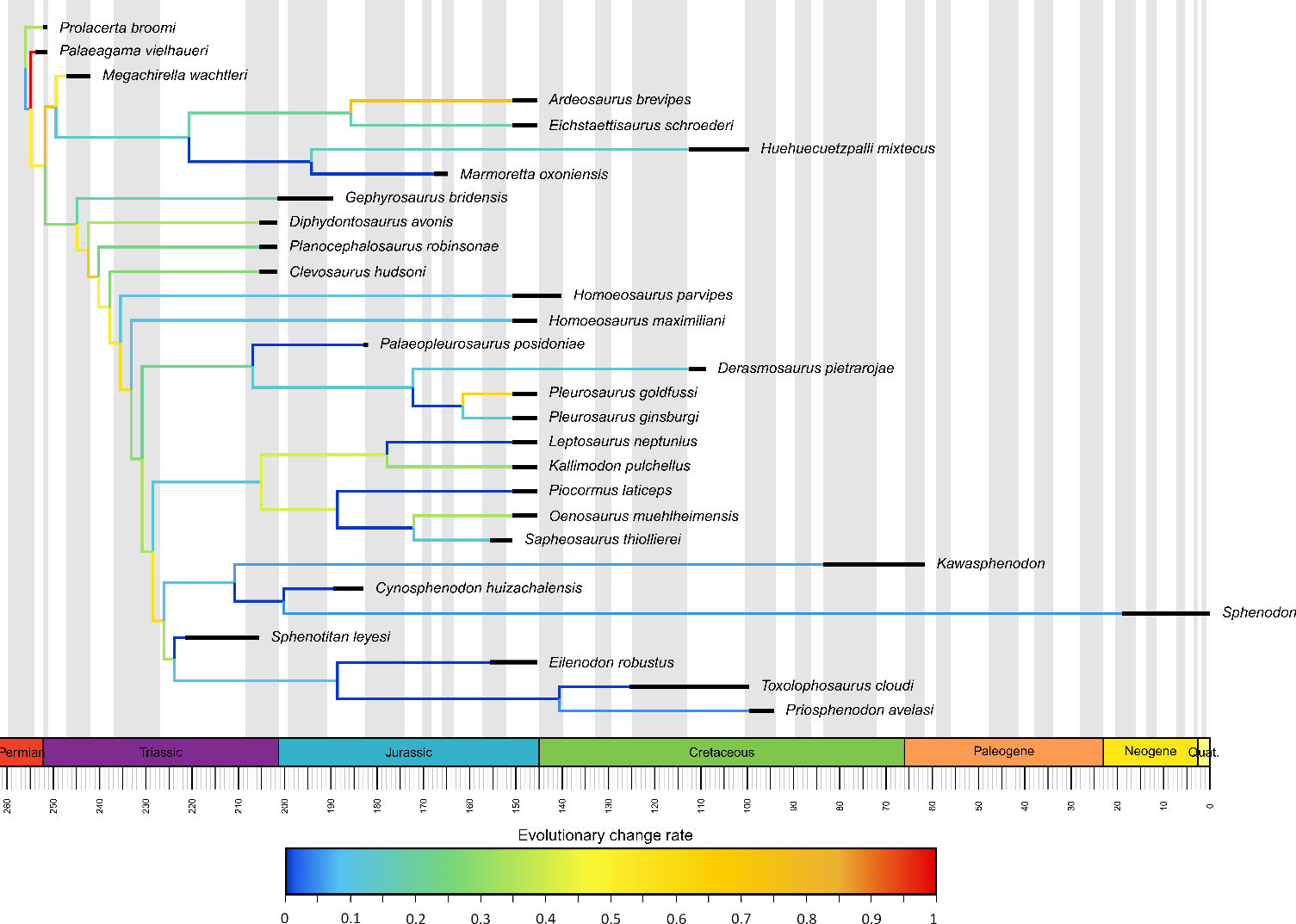


Fig. S8. Evolutionary change rates calculated through character state changes per million years using tip-dates and calculating node ages under the ‘equal’ time-calibration model, excluding *Parvosaurus harudensis*. Rates are log-transformed for easier visualization. Basal sphenodontians consistently show higher rates than derived taxa. Although branch lengths differ significantly from the ‘mbl’ model, both models recover near identical rates when excluding *Parvosaurus harudensis*. Grey and white background stripes indicate age intervals within each geochronological period.


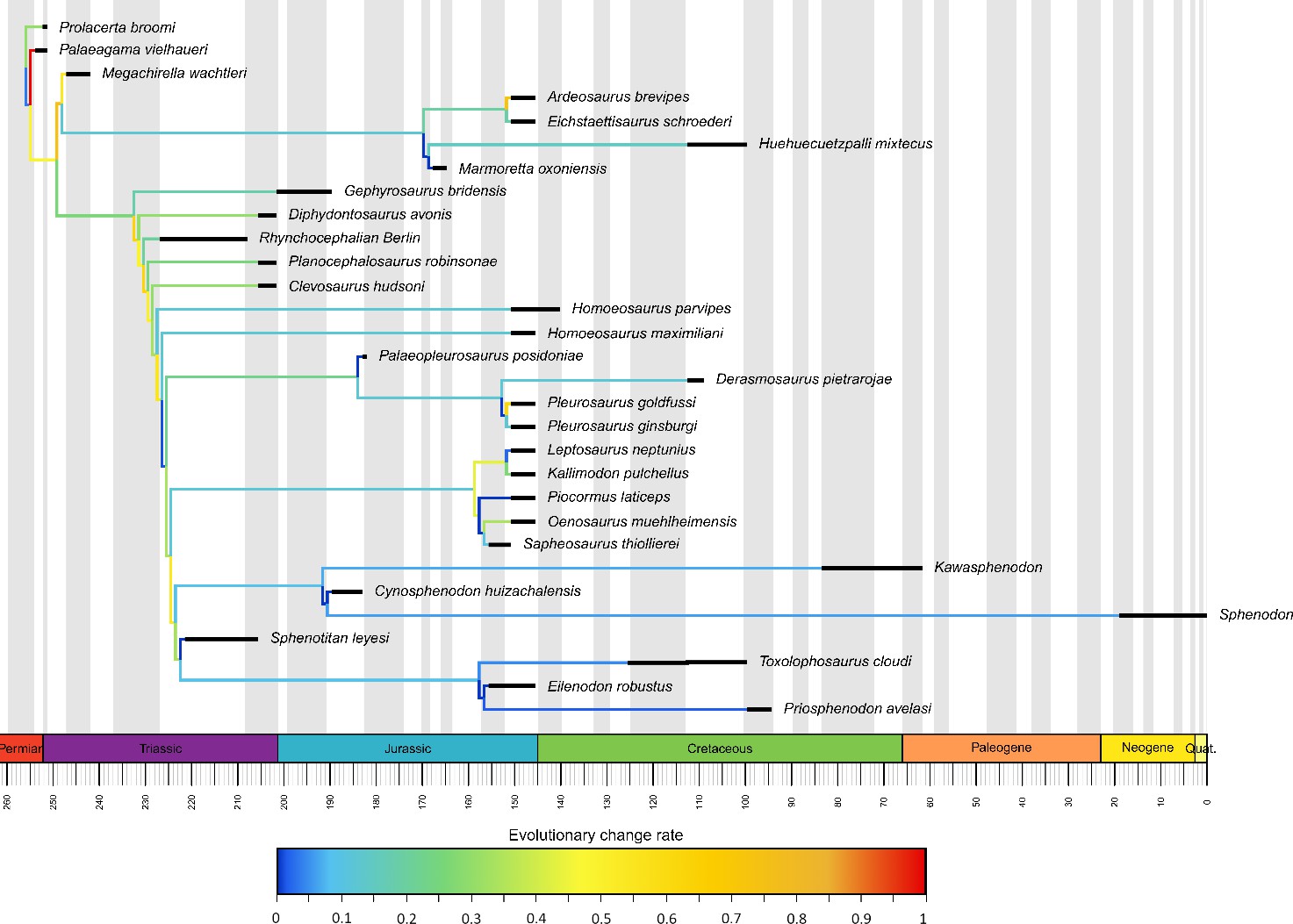


Fig. S9. Evolutionary change rates calculated through character state changes per million years using tip-dates and calculating node ages under the ‘mbl’ time-calibration model, including *Parvosaurus harudensis*. Rates are log-transformed for easier visualization. Basal sphenodontians consistently show higher rates than derived taxa. Recovered branch rates under the ‘mbl’ model stay virtually unchanged when including *Parvosaurus harudensis.* Grey and white background stripes indicate age intervals within each chronostratigraphic age.


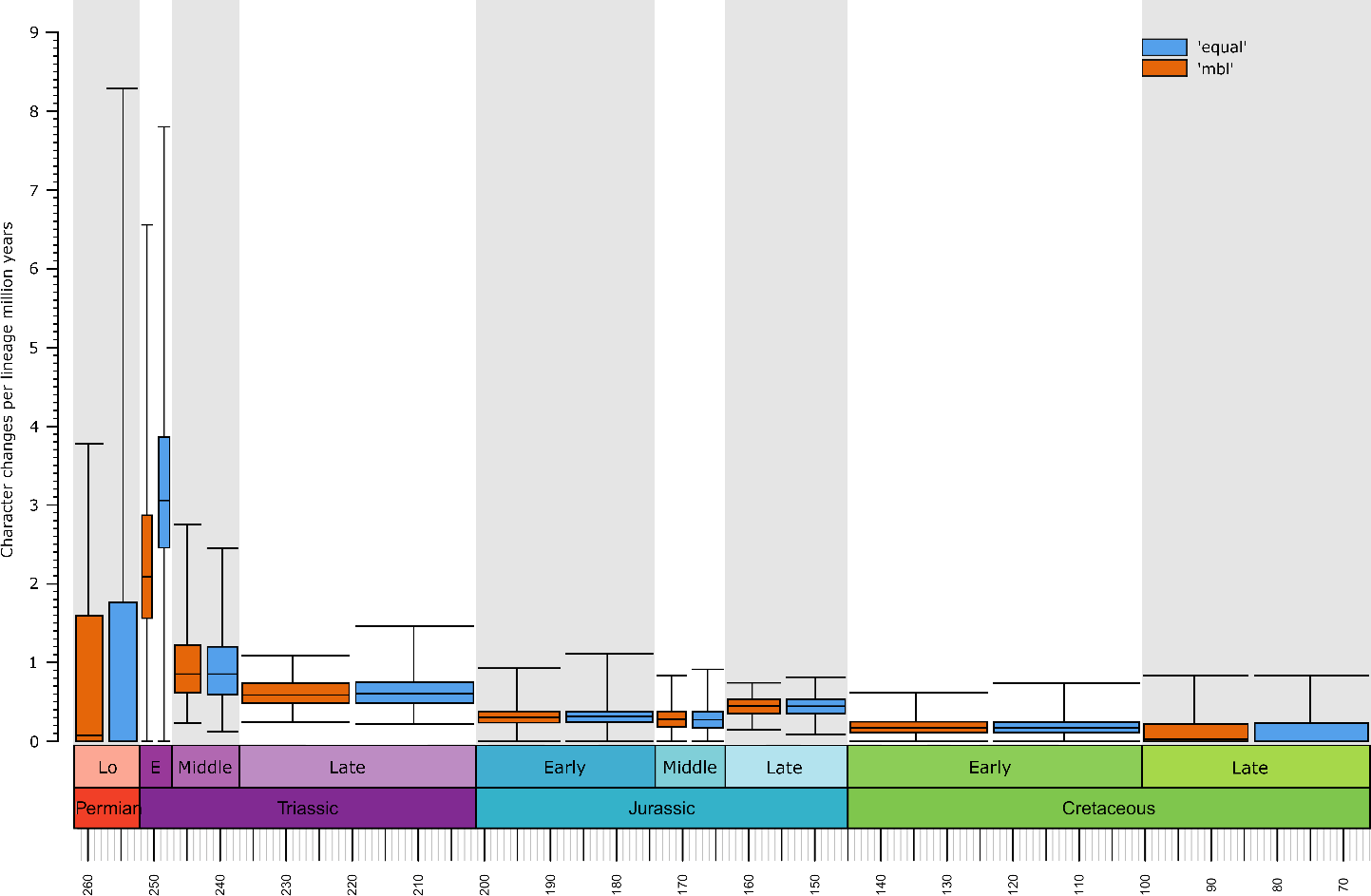


Fig. S10. Sphenodontian evolutionary change rates averaged over geochronological epoch, excluding *Parvosaurus harudensis*. Orange, time-calibration using the ‘mbl’ model; blue, time- calibration using the ‘equal’ model. Although Permian and early Triassic rates generally show lower accuracy, Middle and Late Triassic sphenodontians yield overall higher rates than later sphenodontians in both models. Grey and white background stripes indicate geochronological epochs.


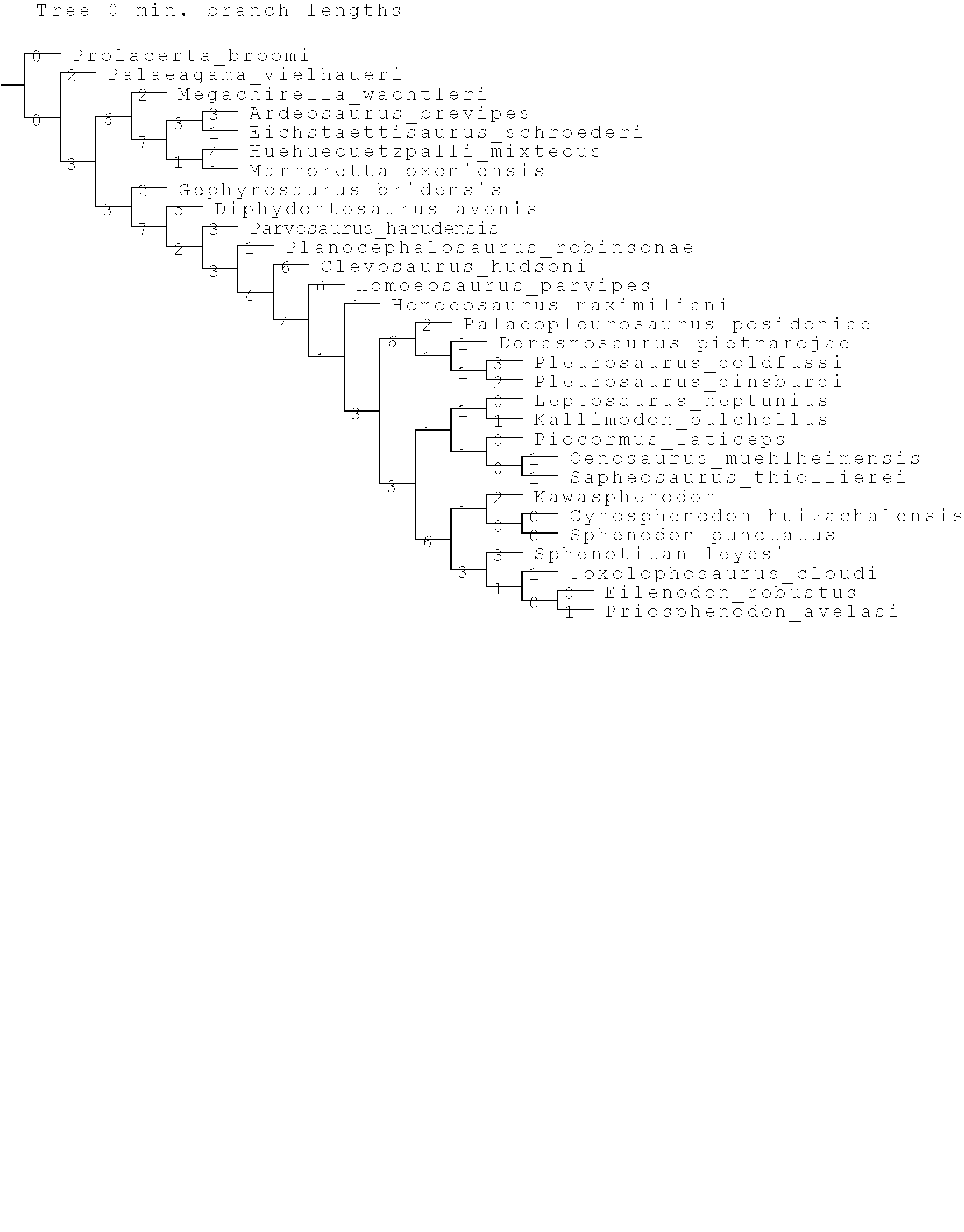


Fig. S11. Maximum parsimony phylogeny showing the number of character changes per branch. Note decreasing number of character state changes on crownward branches.


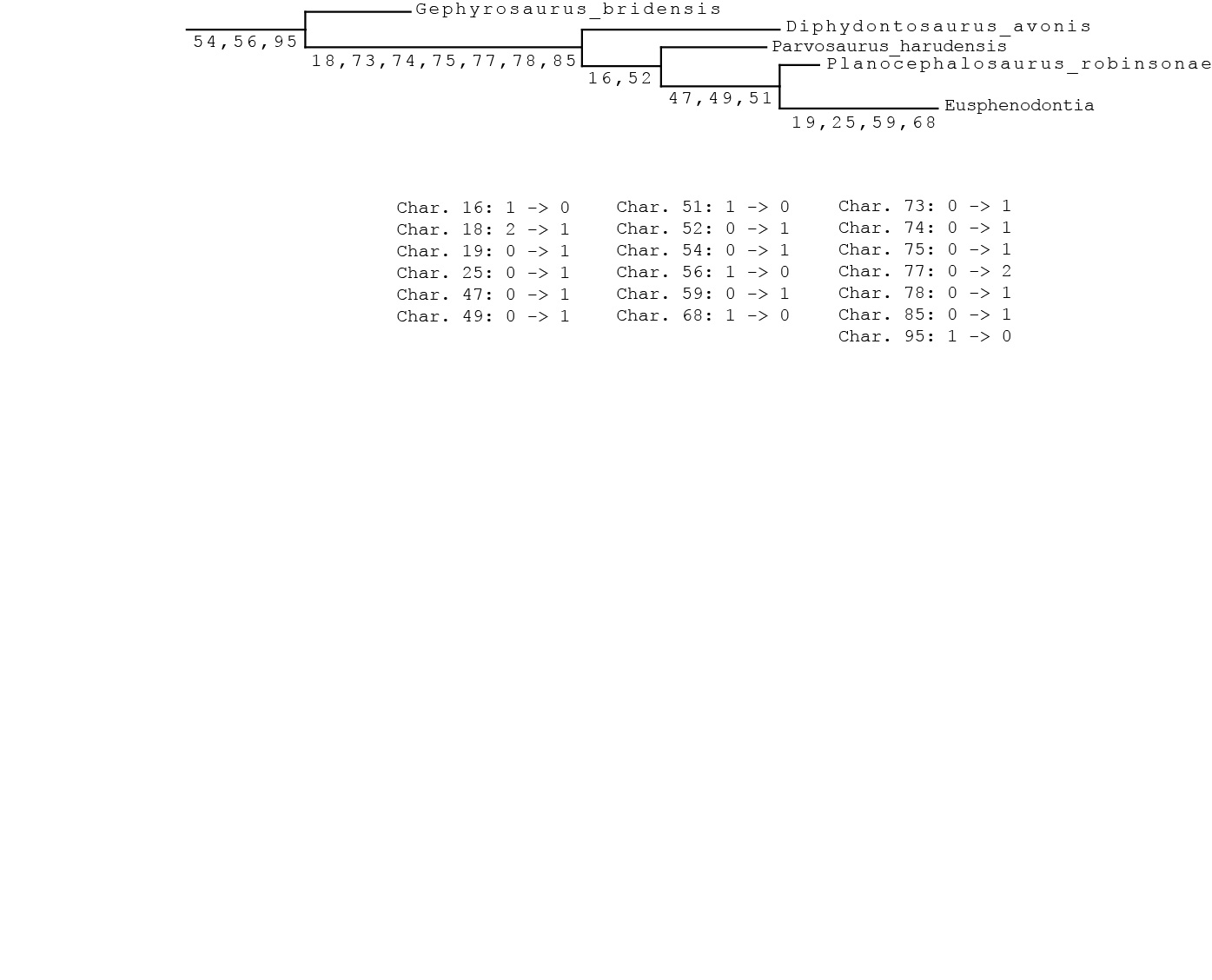


Fig. S12. Mapping of unambiguous synapomorphies at the root of Eusphenodontia mapped in TNT. Note that character numbers in TNT are equivalent with ‘Character number -1’ in the character matrix. Character descriptions: Char. 16, postfrontals, medial forking (state: absent); Char. 18, postfrontals, medial margin, position, relative to parietal (state: dorsal); Char. 19, squamosals, anterior process, lateral surface, facet for postorbital (state: parabolic); Char. 25, frontals, fusion to each other (state: separated); Char. 47, dentary, symphysis, ventral margin, mentonian process (state: present); Char. 49, dentary, symphysis, shape (state: elliptical); Char. 51, dentaries, anterior end, split by Meckelian canal (state: absent); Char. 52, dentaries, subdental shelf (state: absent); Char. 54, dentaries, coronoid process, dorsal expansion (state: present); Char. 56, splenials (state: absent); Char. 59, articular, glenoid cavity, central ridge (state: present); Char. 68, palatine teeth, number of tooth rows (state: one); Char. 73, marginal dentition, posterior teeth series, tooth replacement (state: absent); Char. 74, marginal dentition, hatchling teeth in adults (state: present); Char. 75, marginal dentition, alternating teeth series (present); Char. 77, marginal dentition, posterior teeth series, position, placement relative to jaw bone apical margin (=crista dorsalis) (state: apically); Char. 78, marginal dentition, posterior teeth series, ankylosis to crista dorsalis (apex of labial wall) of dentary (state: present); Char. 85, dentary teeth, posterior teeth series, shape of basal cross section (state: labiolingually compressed); Char. 95, presacral pleurocentra, notochord, persistent in adults (state: present).
